# Aging weakens Th17 cell pathogenicity and ameliorates experimental autoimmune uveitis in mice

**DOI:** 10.1007/s13238-021-00882-3

**Published:** 2021-11-08

**Authors:** He Li, Lei Zhu, Rong Wang, Lihui Xie, Jie Ren, Shuai Ma, Weiqi Zhang, Xiuxing Liu, Zhaohao Huang, Binyao Chen, Zhaohuai Li, Huyi Feng, Guang-Hui Liu, Si Wang, Jing Qu, Wenru Su

**Affiliations:** 1 State Key Laboratory of Ophthalmology, Zhongshan Ophthalmic Center, Sun Yat-Sen University, Guangzhou, 510060 China; 2 State Key Laboratory of Membrane Biology, Institute of Zoology, Chinese Academy of Sciences, Beijing, 100101 China; 3 State Key Laboratory of Stem Cell and Reproductive Biology, Institute of Zoology, Chinese Academy of Sciences, Beijing, 100101 China; 4 Advanced Innovation Center for Human Brain Protection, National Clinical Research Center for Geriatric Disorders, Xuanwu Hospital Capital Medical University, Beijing, 100053 China; 5 CAS Key Laboratory of Genomic and Precision Medicine, Beijing Institute of Genomics, Chinese Academy of Sciences, Beijing, 100101 China; 6 Institute for Stem Cell and Regeneration, Chinese Academy of Sciences, Beijing, 100101 China; 7 Aging Translational Medicine Center, International Center for Aging and Cancer, Xuanwu Hospital, Capital Medical University, Beijing, 100053 China; 8 China National Center for Bioinformation, Beijing, 100101 China; 9 Beijing Institute for Stem Cell and Regenerative Medicine, Beijing, 100101 China; 10 Chongqing Renji Hospital, University of Chinese Academy of Sciences, Chongqing, 400062 China

**Keywords:** aging, experimental autoimmune uveitis, Th17 cell, APCs, single-cell sequencing

## Abstract

Aging-induced changes in the immune system are associated with a higher incidence of infection and vaccination failure. Lymph nodes, which filter the lymph to identify and fight infections, play a central role in this process. However, careful characterization of the impact of aging on lymph nodes and associated autoimmune diseases is lacking. We combined single-cell RNA sequencing (scRNA-seq) with flow cytometry to delineate the immune cell atlas of cervical draining lymph nodes (CDLNs) of both young and old mice with or without experimental autoimmune uveitis (EAU). We found extensive and complicated changes in the cellular constituents of CDLNs during aging. When confronted with autoimmune challenges, old mice developed milder EAU compared to young mice. Within this EAU process, we highlighted that the pathogenicity of T helper 17 cells (Th17) was dampened, as shown by reduced GM-CSF secretion in old mice. The mitigated secretion of GM-CSF contributed to alleviation of IL-23 secretion by antigen-presenting cells (APCs) and may, in turn, weaken APCs’ effects on facilitating the pathogenicity of Th17 cells. Meanwhile, our study further unveiled that aging downregulated GM-CSF secretion through reducing both the transcript and protein levels of IL-23R in Th17 cells from CDLNs. Overall, aging altered immune cell responses, especially through toning down Th17 cells, counteracting EAU challenge in old mice.

**Supplementary Information:**

The online version contains supplementary material available at 10.1007/s13238-021-00882-3.

## INTRODUCTION

With a rapidly expanding elderly population, insights into aging-induced physiological changes will assist in healthy aging. The immune system shows marked changes during aging, which are often manifested as compositional and functional alterations in diverse immune cells (Goronzy and Weyand, 2013; Nikolich-Žugich, 2018). Both T and B cell compartments, main components of the adaptive immunity, show a significant shift from naïve to memory cell types with the expression of a relatively restricted antigen-receptor repertoire during aging (Cambier, 2005; Kogut et al., 2012; Nikolich-Žugich, 2018). With respect to innate immunity, the impact of aging on them often varies across cell types, cell states, and tissue contexts (Montgomery and Shaw, 2015). Despite steady progress in understanding the influence of aging on the immunological system, the underlying mechanisms still require further investigation (Nikolich-Žugich, 2018).

Age-related deterioration of the immune system is associated with increased susceptibility to infections and vaccination failure in older people (Gavazzi and Krause, 2002; Pinti et al., 2016; Goronzy and Weyand, 2017). However, there have been insufficient studies about the impact of aging on autoimmune diseases and their results are inconsistent. Although several studies have proposed that older people display greater autoimmunity characterized by increased serum levels of autoantibodies and accumulation of autoreactive T cells due to checkpoint failures during aging (Goronzy and Weyand, 2012; Müller and Pawelec, 2015), the incidence of many autoimmune diseases is lower among the elderly (Cooper and Stroehla, 2003). Indeed, most autoimmune diseases, such as systemic lupus erythematosus and multiple sclerosis, rarely occur in the elderly and, when they occur, may present with milder symptoms than in younger individuals (Cooper and Stroehla, 2003; Rovenský and Tuchynová, 2008).

Autoimmune uveitis (AU) is an autoimmune disease of the central nervous system (CNS) characterized by immune-mediated inflammatory disorders of the eye and is among the leading causes of visual impairments and blindness (de Smet et al., 2011; Thorne et al., 2016). The possible pathogenic mechanisms of AU have been gradually revealed based on studies of experimental autoimmune uveitis (EAU), a widely accepted animal model of AU (Pennesi *et al.*,^2003^). However, how aging impacts AU remains to be studied. Clinically, AU predominantly affects people of working age (20 to 50 years of age), and it is hardly observed in the elderly (Papotto et al., 2014). Although a negative relationship between aging and the incidence of AU is commonly found, the underlying mechanism is not clear.

Lymph nodes (LNs) are secondary lymphoid organs that facilitate the interaction among various immune cells (Gasteiger et al., 2016), and they are critical for the regulation of adaptive immune reaction and pathogen clearance (Gasteiger et al., 2016). LNs are also responsible for peripheral tolerance (Samy et al., 2005). However, once the tolerance is broken for some pathological reasons, autoantigens are presented to autoreactive T cells and B cells in LNs (Muñoz et al., 2010; Mochizuki et al., 2013). The interaction with autoantigens results in the proliferation and activation of autoreactive T cells and B cells, as well as the formation of germinal center (GC) in LNs, thus initiating autoimmune diseases (Muñoz et al., 2010; Yu et al., 2015; Theofilopoulos et al., 2017). During aging, LNs undergo structural and functional alterations (Turner and Mabbott, 2017). Specifically, altered structural organization, defective cell trafficking, and delayed germinal center reaction have been observed in aged LNs (Richner et al., 2015a; Turner and Mabbott, 2017). These changes contribute to impaired adaptive immunity, and account for a higher risk of infections and vaccination failure in older people (Richner et al., 2015b; Tabibian-Keissar et al., 2016). However, the impact of aging on autoimmune responses, especially those associated with LNs, has not been defined.

Single-cell RNA sequencing (scRNA-seq) is a powerful tool for analyzing samples with complex cellular constituents (Grün and van Oudenaarden, 2015), and its application has facilitated the understanding of the pathogenesis of many diseases (Rantalainen, 2018; Paik et al., 2020; Luo et al., 2020). scRNA-seq has been applied to profile immune cells of LNs, including axillary, brachial, and inguinal LNs, in various health and disease conditions (Rodda et al., 2018; Blecher-Gonen et al., 2019; Veerman et al., 2019). However, the single-cell transcriptomes of aged LNs and LNs in autoimmune conditions have not been delineated. Cervical draining LNs (CDLNs) are the main draining LNs of the central nervous system (CNS) and contribute to the pathogenesis of autoimmune diseases in CNS (Louveau et al., 2018), which are therefore ideal for exploring immune changes during EAU. Immune cell atlases of aged CDLNs and CDLNs during EAU will facilitate the understanding of the impact of aging on EAU and may provide insights into the interaction between aging and a wider range of autoimmune diseases.

In this study, we used scRNA-seq and flow cytometry to map the immune cell atlas of CDLNs from young and old mice with or without EAU and observed that aging-induced marked changes in the transcriptomes of various immune cell types in CDLNs. In response to EAU challenge, old mice exhibited alterations in immune cell compositions and functions different from those of young mice, which may explain their difference in EAU symptoms. Within this process, our study highlighted that aging weakened the pathogenicity of T helper 17 (Th17) cells, reflected in their reduced GM-CSF secretion. Coculturing antigen-presenting cells (APCs) with these restrained Th17 cells resulted in decreased IL-23 secretion by APCs, which may further impair APCs’ capacity of promoting the pathogenicity of Th17 cells. Meanwhile, aging downregulated GM-CSF secretion of Th17 cells by reducing the mRNA and protein levels of IL-23R in Th17 cells. Overall, aging altered immune responses to EAU challenge, especially through toning down the pathogenic Th17 cells, thus ameliorating EAU development in old mice.

## RESULTS

### Aging induces complicated and extensive changes in the immune profile of CDLNs

**Figure 1:**
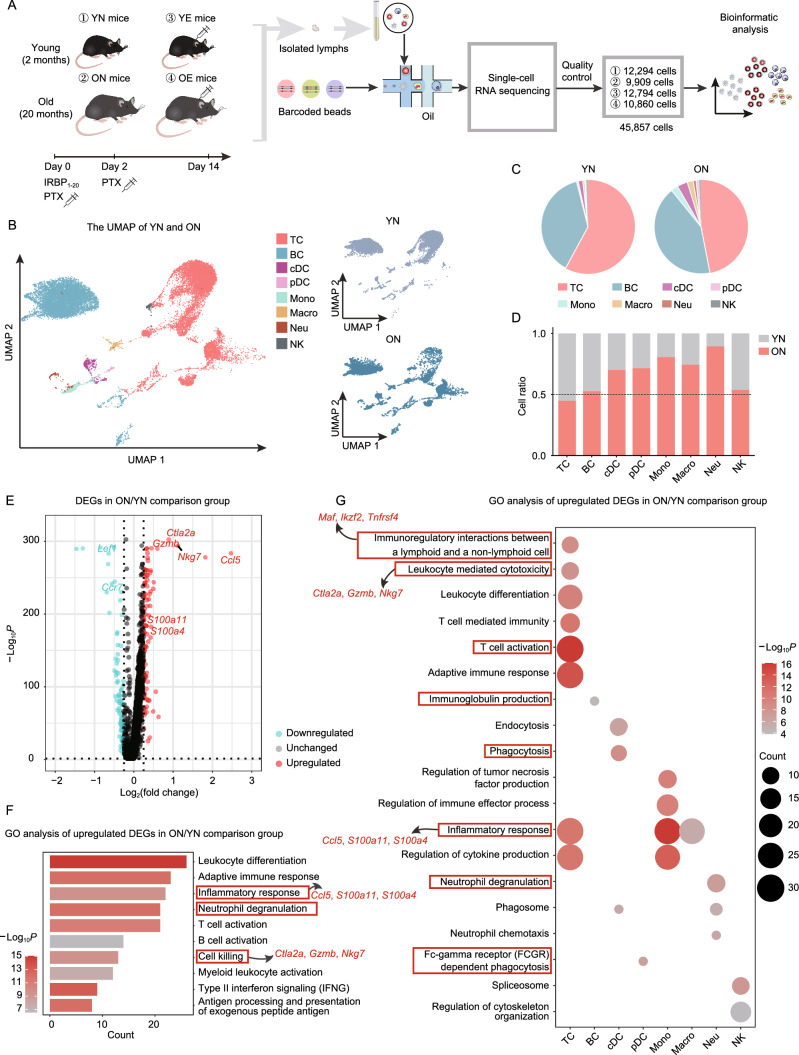
Study design and analysis of aging-induced alterations in immune cell profiles of CDLNs. (A) Schematic of the experimental design for single-cell RNA sequencing. CDLNs were harvested from young (Y) and old (O) mice without (N) or with (E) EAU. Each sample included three mice. Samples were processed via scRNA-seq by using the 10x Genomics platform. IRBP_1-20_: Interphotoreceptor retinoid-binding protein 1-20; PTX: Pertussis toxin. (B) UMAP plot showing clusters of immune cell subsets. (C) Pie charts showing relative cluster abundance of immune cell subset in YN and ON mice. (D) Bar chart showing the cell ratios of immune cell subsets in YN and ON mice derived from scRNA-seq data. (E) Volcano plot showing upregulated and downregulated DEGs of all immune cell types in the ON/YN comparison group. Red and blue dots indicate upregulated and downregulated DEGs in ON group compared to YN group, respectively. (F and G**)** Representative GO terms and KEGG pathways enriched in upregulated DEGs of total immune cells (F) or immune cell subsets (G) in the ON/YN comparison group

To probe the impact of aging on the immune profile of CDLNs and to fully explore the interaction between aging and autoimmunity, we generated scRNA-seq data from CDLNs of young normal mice (YN), old normal mice (ON), young mice with EAU (YE) and old mice with EAU (OE) (Fig. 1A). EAU was induced by retinal antigen interphotoreceptor retinoid-binding protein 1–20 (IRBP_1–20_), which is the most common uveitogenic retinal protein in mice (Caspi et al., 2008). Single-cell suspensions were harvested from isolated CDLNs and converted to barcoded scRNA-seq libraries via 10x Genomics for further analysis. We combined the CellRanger software with the Seurat package for initial processing, quality control, and analysis of the sequencing data. After quality control, we obtained a total of 45,857 high-quality cells (Fig. 1A).

We first examined the general impact of normal aging on the immune profile of CDLNs. We clustered cells from YN and ON mice and identified eight immune cell lineages, including T cells (TC), B cells (BC), conventional dendritic cells (cDC), plasmacytoid dendritic cells (pDC), monocytes (Mono), macrophages (Macro), neutrophils (Neu), and natural killer cells (NK), according to classical markers and other uniquely upregulated genes (Figs. 1B, S1A, and S1B). As shown in Fig. 1C, T cells and B cells are the primary cellular constituents of CDLNs from both YN and ON mice. During aging, the proportion of myeloid cells increased (Fig. 1D). To identify the global gene signatures of CDLN cells associated with normal aging, we identified the differentially expressed genes (DEGs) of all cell types identified in CDLNs from ON and YN mice and conducted Gene Ontology (GO) analysis of these genes (Fig. 1E, 1F; Table S1A). For example, DEG analysis revealed that the naïve phenotype-associated genes *Lef1* and *Ccr7* were expressed less in ON mice than in YN mice (Fig. 1E; Table S1A). Upregulated DEGs in ON mice were enriched in inflammation-related pathways (annotated as neutrophil degranulation and inflammatory response, the latter harboring DEGs such as *Ccl5*, *S100a11*, *S100a4*) and cytotoxic pathway (annotated as cell killing, associated with DEGs including *Ctla2a*, *Gzmb*, and *Nkg7*) (Patil et al., 2018) (Fig. 1E, 1F; Table S1A). Genes downregulated in ON mice were enriched for pathways such as peptide biosynthetic and amide biosynthetic processes (Fig. S1C). These DEG and GO analyses collectively indicated inflammatory states of aged CDLNs.

We then delineated the aging-induced alterations in the cell type-specific transcriptomes (Table S1B–I). In the context of aging, upregulated DEGs of T cells were enriched in T cell activation, inflammatory response, immunoregulatory interactions, and leukocyte mediated cytotoxicity pathways (Figs. 1G and S1D). In B cells, the immunoglobin production pathway was upregulated (Fig. 1G). Among myeloid cells, cDC and pDC from ON mice highly expressed genes that were enriched in phagocytosis pathways (annotated as phagocytosis or Fc-gamma receptor (FCGR)-dependent phagocytosis) (Fig. 1G), indicating enhanced phagocytosis capacity with diminished antigen-presenting capacity (Savina and Amigorena, 2007). In monocytes and macrophages, we observed aging-upregulated biological pathways related to inflammatory responses, suggesting a bias towards the inflammatory phenotypes (Fig. 1G). In neutrophils, the upregulated neutrophil degranulation pathway suggested that these cells were in active states during aging (Fig. 1G). As for downregulated genes of each major immune cell type in ON mice, pathways such as SRP-dependent cotranslational protein targeting to membrane and amide biosynthetic process were enriched (Fig. S1E). Additionally, downregulated aging DEGs in NK cells were involved in cytotoxicity-related pathways, which was consistent with reduced natural killer cell cytotoxicity during aging as previously reported (Solana et al., 2006) (Fig. S1E). Overall, we have developed a global immune profile of aged CDLNs. Immune cells from aged CDLNs are prone to exhibit inflammatory phenotypes, with each immune cell type showing their respective compositional and functional alterations.

### Aging induces compositional and functional changes of T cell and B cell compartments in CDLNs

LNs mainly consist of T cells and B cells, where they are activated by encountering antigen (Gasteiger et al., 2016). T cells and B cells are the main components of adaptive immunity; therefore, they have been the focus of studies on aging and immunity (Goronzy and Weyand, 2019; Elyahu and Monsonego, 2021). Thus, we next examined aging-induced alterations in T cells and B cells.

**Figure 2:**
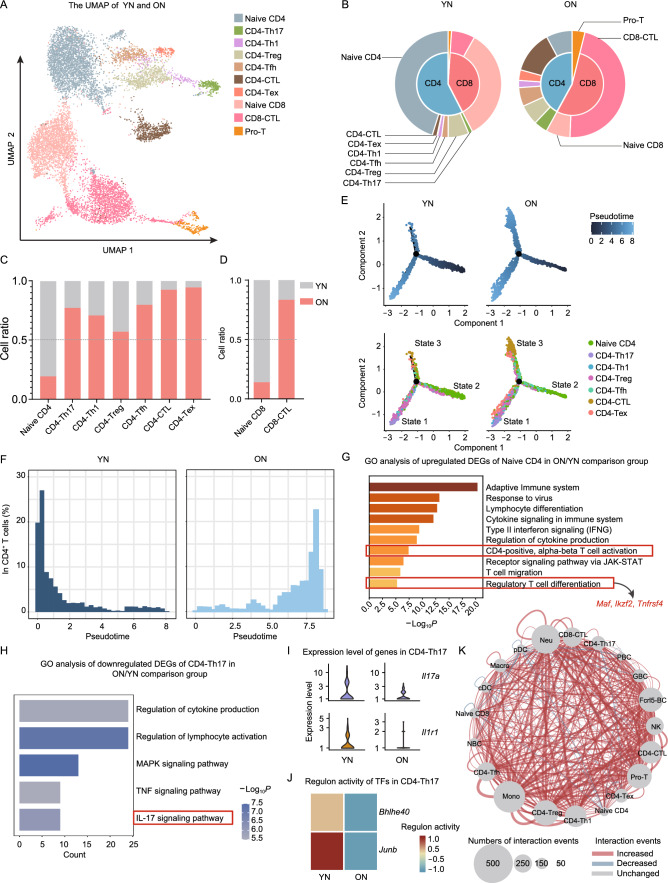
Aging-induced alterations in T cell subsets. (A) UMAP plot showing subclusters of T cell subsets. (B) Pie charts showing relative cluster abundance of T cell subsets in the YN and ON mice. (C and D) Bar charts showing the cell ratios of CD4^+^ T cell subsets (C) or CD8^+^ T cell subsets (D) in YN and ON mice derived from scRNA-seq data. (E) Pseudotime trajectory analysis corresponding to the differentiation of effector CD4^+^ T cells from naïve CD4^+^ T cells. Cells are colored by pseudotime (top) or cell type (bottom), as indicated. (F) Percentages of CD4^+^ T cells along the pseudotime for YN and ON mice. (G and H) Representative GO terms and KEGG pathways enriched in upregulated DEGs of naïve CD4^+^ T cells (G) or Th17 cells (H) in the ON/YN comparison group. (I) Violin plots of expression of *Il17a* and *Il1r1* in Th17 cells of YN and ON mice. (J) Heatmap of relative regulon activity of *Bhlhe40* and *Junb* from Th17 cells in the YN and ON mice. (K) Network plots showing the changes in ligand-receptor interaction events between different cell types in the YN/ON comparison group. Cell-cell communication is indicated by the connected line. The thickness of the lines is positively correlated with the number of ligand-receptor interaction events

We identified ten subtypes of T cells, including naïve CD4^+^ T cells (naïve CD4), naïve CD8^+^ T cells (naïve CD8), CD4^+^ T cells with cytotoxic activity (CD4-CTL), CD8^+^ T cells with cytotoxic activity (CD8-CTL), T helper 17 cells (CD4-Th17), regulatory T cells (CD4-Treg), T follicular helper cells (CD4-Tfh), T helper 1 cells (CD4-Th1), proliferative T cells (Pro-T), and exhausted T cells (CD4-Tex) (Figs. 2A, S2A, and S2B). Naïve T cells dominated in T cell compartments in YN mice, whereas CD4-CTL and CD8-CTL dominated in T cell compartments in ON mice (Fig. 2B). The proportions of most T cell subsets, except for naïve T cells, increased in ON mice compared to YN mice (Fig. 2C and 2D). The proportion of naïve T cells decreased in ON mice, possibly due to their ongoing differentiation into effector and memory T cells during aging as previously reported (Frasca and Blomberg, 2011). To clarify this hypothesis, we conducted pseudotime analysis and observed a pseudotemporal path started from naïve CD4 to effector T cells. Compared to YN mice, ON mice showed more effector T cells, including CD4-Th17, CD4-Th1, CD4-Treg, CD4-Tfh, CD4-CTL, and CD4-Tex in state 1, as well as more CD4-CTL in state 3, but less naïve CD4 T in state 2 (Fig. 2E). This result indicated enhanced differentiation of naïve CD4 into effector T cells in ON mice (Figs. 2F, S2C, and S2D). Similarly, among CD8^+^ T cells, less naïve CD8 in the initiation and more CD8-CTL in the end of the pseudotemporal path were in ON mice than in YN mice, indicating enhanced differentiation of naïve CD8 into CD8-CTL in ON mice (Fig. S3A–D).

GO analysis revealed that the upregulated DEGs of naïve CD4 from ON mice were enriched in T cell activation pathway and regulatory T cell differentiation pathway, including *Maf*, *Ikzf2*, and *Tnfrsf4* (Figs. 2G and S3E; Table S1J). Cytotoxic genes (*Ctla2a* and *Nkg7*) were upregulated in naïve CD8 from ON mice (Fig. S3F; Table S1Q). Upregulated DEGs in naïve CD8 from ON mice were also enriched in the lymphocyte differentiation pathway (Fig. S3G). These results indicated that naïve T cells in aged CDLNs differentiated actively and tended to exhibit a mix of inflammatory, regulatory, and cytotoxic phenotypes. Since Th17 cells are a T cell subset actively involved in AU and EAU (Harrington et al., 2005), we next analyzed their aging-induced transcriptional changes. GO analysis revealed downregulated IL-17 signaling pathway in CD4-Th17 from ON mice compared to YN mice (Fig. 2H). Correspondingly, the expression of *Il17a* was lower in ON mice compared to YN mice (Figs. 2I and S3H; Table S1K). Moreover, the expression of *Il1r1*, a pathogenic Th17 cell-related gene (Yasuda et al., 2019), as well as the regulon activity of *Bhlhe40* and *Junb*, two pathogenic transcriptional factors (TFs) of Th17 cells (Lin et al., 2014; Hasan et al., 2017), were all lower in ON mice than in YN mice (Figs. 2I, 2J, and S3I; Table S2). These results may indicate that aging induces functional impairment in Th17 cells. Among other T cell subsets, T cell activation and inflammation-related pathways were generally upregulated in ON mice (Fig. S4A; Table S1J–S). We also observed downregulated pathways such as peptide biosynthetic process and cytoplasmic translation, in most T cell subsets (Fig. S4B; Table S1J–S). Thus, aging induced various alterations in different T cell subsets. Specifically, active differentiation of naïve T cells, impaired function of Th17 cells were observed in aged CDLNs.

As for the B cell compartments, we identified four B cell subsets, i.e., naïve B cells (NBC), germinal B cells (GBC), plasma B cells (PBC), and Fcrl5-positive B cells (Fcrl5-BC) (Fig. S5A–C). Increased proportion of Fcrl5-B cells, germinal B cells, and plasma B cells were observed in ON mice compared to YN mice (Fig. S5D). Pseudotime analysis showed that a pseudotemporal path started from naïve B cells to effector B cells. We observed that more plasma B cells and Fcrl5-B cells were in state 1 and more germinal B cells were in state 2 in ON mice compared to YN mice, indicating enhanced differentiation from naïve B cells to effector B cells (Fig. S5E–H). The relatively large proportion of naïve B cells may explain that although their proportion just slightly decreased in ON mice, active differentiation from naïve subsets to effector subsets still existed in ON mice. We then conducted GO analysis to explore functional changes in B cell subsets. Aging upregulated the antigen processing and presentation pathway in naïve B cells and the immunoglobulin production pathway in plasma B cells, as well as downregulated pathways such as peptide biosynthetic process in all B cell subsets (Fig. S6A and S6B; Table S1T–W). Thus, the two main functions of B cells, namely, antigen processing and presentation, as well as immunoglobulin production, might be preserved and even enhanced in B cells of the elderly.

We further explored the cell-cell interaction among T cell and B cell subsets, along with other major immune cell types (Table S5). The cell-cell interaction events were integrally increased among different cell types during aging (Fig. 2K). Activation and differentiation of T cells require co-stimulation from DC (driven by CD28 and its ligand CD86) (Levine et al., 1995), whereas activation and differentiation of B cells require co-stimulation provided by T cells (driven by CD40 and its ligand CD40LG) (Armitage et al., 1992). We observed enhanced CD28-CD86 interaction between naïve T cells and cDC, accompanied by enhanced CD40-CD40LG interaction between germinal or naïve B cells and Tfh cells in ON compared to YN (Fig. S6C). APRIL, a proliferation-inducing ligand, plays an important role in B cell differentiation (Shabgah et al., 2019; Yeh et al., 2020). We found that the APRIL signaling network was more potent in ON mice than in YN mice (Fig. S6D). These results partially explained the active differentiation from naïve to effector cells and the proportional change of T cell and B cell subsets in old mice. In addition, the interaction of TGF-β (encoded by *Tgfb1*) and its receptors between Tregs and several other immune cells was among the increased interaction events led by aging (Fig. S6E). TGF-β is the main molecule that mediates the immunosuppressive ability of Tregs (Liu et al., 2018). Thus, Tregs in ON mice might have enhanced immune-repressing capacity compared to those of YN mice as previously reported (Elyahu et al., 2019).

In summary, our single-cell data revealed reduced naïve T cells, and increased effector cells in both T cell and B cell compartments including Th1, Th17, and plasma cells in ON mice. These results indicated that old mice may behave differently when experiencing autoimmune diseases compared to young individuals. Functionally, GO and DEG analyses of T cells indicated that naïve T cells differentiate actively and prefer to exhibit a mixture of inflammatory, regulatory, and cytotoxic phenotypes whereas the function of Th17 cells might be impaired in old mice. B cells from aging CDLNs showed preserved antigen processing and presentation as well as immunoglobin production capacity. In addition, cell-cell communications, including ligand-receptor pairs related to T and B cell activation and immune repressing ability of Tregs, are generally enhanced in old mice.

### Aging mitigates EAU symptoms and alters immune cell response to EAU challenge

**Figure 3:**
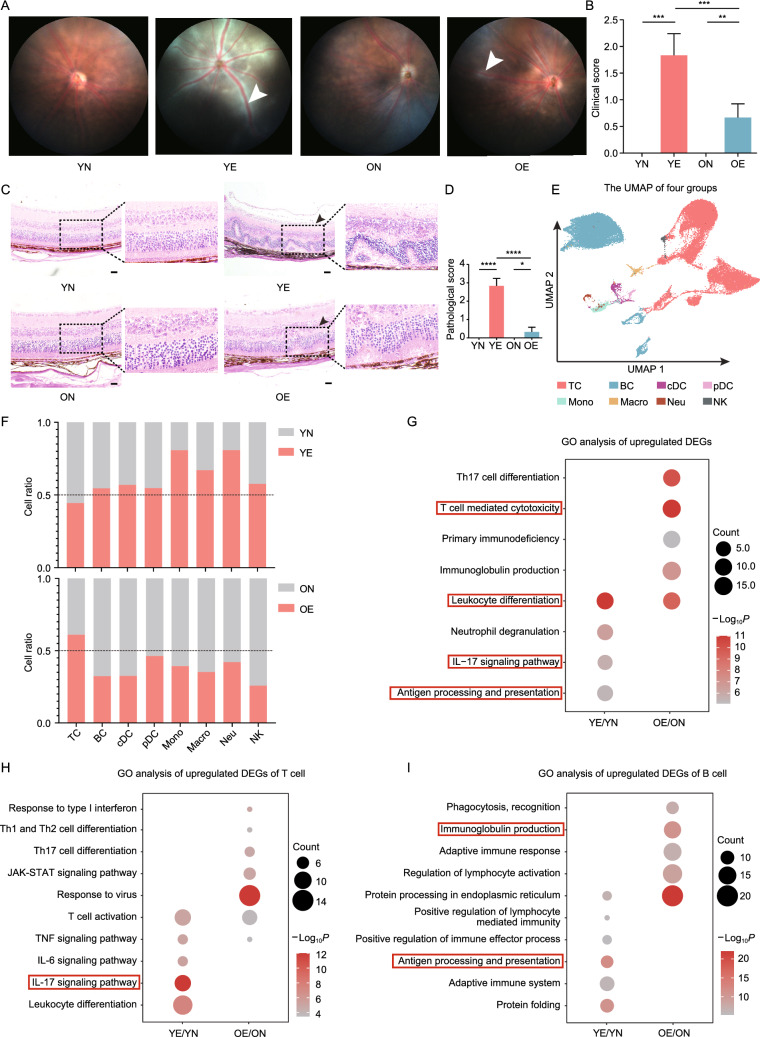
Milder EAU symptoms and altered immune cell responses to EAU challenge induced by aging. (A) Representative fundus imaging of YN, YE, ON, and OE obtained 14 days after immunization. White arrowheads indicate inflammatory exudation and vascular deformation. (B) Bar plot showing clinical scores of the four groups. Each group contains six mice. The values represent the mean ± SD. Significance was determined using two-way ANOVA. **P < 0.01, ***P < 0.001. (C) Histopathological images of hematoxylin and eosin-stained eye sections of the four groups. Black arrowheads indicate magnified area with infiltration of inflammatory cells and retinal folding. Scale bars, 20 mm. (D) Bar plot showing pathogenic scores of the four groups. Each group contains six mice. The values represent the mean ± SD. Significance was determined using two-way ANOVA. *P < 0.05, ****P < 0.0001. (E) UMAP plot showing clusters of immune cell subsets. (F) Bar chart showing the cell ratios of immune cell subset in the YE/YN and OE/ON comparison groups derived from scRNA-seq data. (G–I**)** Representative GO terms and KEGG pathways enriched in upregulated DEGs of total immune cells (G), T cells (H), or B cells (I) in the YE/YN and OE/ON comparison groups

AU was hardly observed in elderly people (Papotto et al., 2014). To elucidate this phenomenon and further analyze the relationship between aging and AU, we next evaluated aging-associated alterations in autoimmune responses during EAU. We first evaluated EAU symptoms in young and old mice by photographing the eye with a fundus camera on day 14 and grading the disease severity according to the published EAU clinical grading scale (Chen and Caspi, 2019a). EAU in the fundus manifested as multiple chorioretinal lesions and/or infiltrations (Fig. 3A and 3B; Table S6). Hemotoxylin and eosin (H&E) staining of sectioned eyeballs showed inflammatory infiltration and retinal folding in EAU lesions (Fig. 3C and 3D; Table S6). EAU challenge enlarged the CDLNs from both young and old mice (Fig. S7A). Intriguingly, OE mice exhibited milder EAU symptoms, defined by lower clinical scores and pathological scores, compared to YE mice (Fig. 3B and 3D; Table S6). This divergence in the EAU severity between young and old mice was in line with the low incidence rate of AU in the elderly population (Papotto et al., 2014).

To delineate the aging-induced alterations in immune cell response during EAU, CDLN cells from YN, YE, ON, and OE mice were subjected to the single-cell analysis, and major immune cell clusters were identified according to classical markers (Figs. 3E, S7B, and S7C). To demonstrate aging-induced changes in cell composition dynamics in response to EAU, we compared the proportion of each immune cell type between YE and YN mice, in comparison to the change between OE and ON mice (Fig. 3F). Most myeloid cells were increased after EAU development in young mice, but decreased in old mice (Fig. 3F). In contrast, we observed a slight increase in the T cell proportion in OE mice (Fig. 3F). These results showed divergent cell composition changes in response to EAU challenge between young and old mice.

GO analysis showed that total immune cells upregulated the leukocyte differentiation pathway and downregulated the negative regulation of immune system process in response to EAU challenge in both young and old mice (Figs. 3G and S7D; Tables S3A and S4A). When each type of immune cell was analyzed individually, those involved in innate immunity showed comparable upregulated pathways in young and old mice in response to EAU challenge, and most of them were related to enhanced inflammation (Fig. S8A; Tables S3B–I and S4B–I). In contrast, in response to EAU challenge, the IL-17 signaling pathway and the antigen processing and presentation pathway were upregulated in young mice only, whereas T cell-mediated cytotoxicity was upregulated in old mice only (Fig. 3G). The antigen processing and presentation pathway was upregulated in response to EAU challenge in DC from young mice but was barely upregulated in DC from old mice (Fig. S8A). In T cells, the IL-17 signaling pathway was upregulated in YE mice, but not in OE mice (Fig. 3H). This result indicated impaired function of Th17 cells during aging. In B cells, the antigen processing and presentation pathway was enriched in YE mice, whereas the immunoglobulin production pathway was upregulated in OE mice (Fig. 3I). GO analysis of downregulated DEGs showed that negative regulation of immune system process was downregulated in T cells from old mice alone, whereas negative regulation of B cell activation pathway was specifically downregulated in B cells from young mice during EAU (Fig. S8B). In summary, although similar responses in total immune cells are presented in young and old mice in response to EAU challenge, aging clearly induces additional alterations different in T cells and B cells in the EAU model.

### Aging induces altered responses of T cell and B cell subsets during EAU

**Figure 4:**
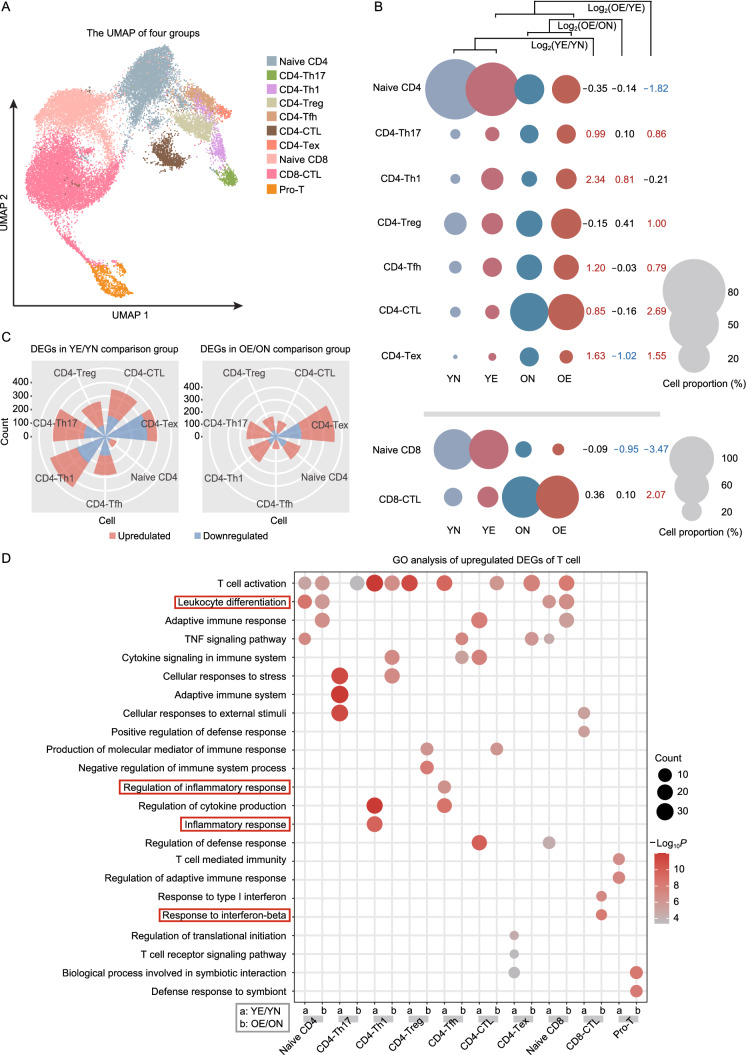
Altered T cell subset responses to EAU challenge. (A) UMAP plot showing clusters of T cell subsets. (B) The fold change (FC) of cell ratios in different T cell subsets across the four groups (YN, YE, ON, and OE). The numbers on the right indicate the Log_2_FC values of the cell ratios (YE/YN, OE/ON, and OE/YE). (C) Wind rose diagrams showing the numbers of upregulated and downregulated DEGs of CD4^+^ T cells in the YE/YN and OE/ON comparison groups. (D) Representative GO terms and KEGG pathways enriched in upregulated DEGs of T cell subsets in the YE/YN and OE/ON comparison groups

Our data above showed that aging-induced distinct T cell and B cell responses to the EAU challenge. Thus, we next investigated the impact of aging on the response of T cell and B cell subsets to EAU challenge. T cells from YE and OE mice were also clustered into ten subsets, similar to the classification of T cells from YN and ON mice (Figs. 4A and S9A). We then explored different compositional changes of T cell subsets in response to the EAU challenge with respect to age (Fig. 4B). Most CD4^+^ T cell subsets increased after EAU development in young mice, including CD4-Th1 and CD4-Th17 that could promote EAU pathogenesis (Luger et al., 2008), CD4-Tfh with B cell regulating function (Crotty, 2011) (Fig. 4B). However, this expansion was refrained in old mice (Fig. 4B). Resulted from divergent baseline proportions, although aging refrained most CD4^+^ effector T cell subsets from extensive expansion in response to EAU challenge, the proportion of most CD4^+^ effector T cell subsets, including CD4-Th17, CD4-Treg, and CD4-Tfh, was still higher in OE mice than in YE mice (Fig. 4B). The proportion of CD8^+^ T cell subsets did not increase in response to EAU challenge in both young and old mice (Fig. 4B), suggesting their relatively weaker role in EAU development compared to CD4^+^ T cells. Thus, we then focused on the aging-induced gene expression alterations in the CD4^+^ T cell subset in response to EAU challenge.

After EAU development, 2,242 and 1,611 DEGs were detected in CD4^+^ T cells of young mice and old mice, respectively (Fig. 4C; Tables S3J–P and S4J–P). In young mice, DEGs in CD4-Th1, CD4-Th17 and CD4-Tex accounted for most DEGs, whereas DEGs in CD4-Tex account for most DEGs in old mice (Fig. 4C). This result suggested that Th1 and Th17 cells played an important role in EAU development in young mice, but their effects were compromised in old mice. We also conducted GO analysis on all T cell subsets (Figs. 4D and S9B; Tables S3J–S and S4J–S). Naïve T cells, including naïve CD4 and naïve CD8, showed upregulated leukocyte differentiation pathway in both young and old mice during EAU (Fig. 4D). In addition, pathways related to inflammation response were only upregulated in effector T cells (including CD4-Th1 and CD4-Tfh) of YE mice, while response to interferon-beta was enriched in CD8-CTL, exclusively in old mice (Fig. 4D). We also observed downregulated DEGs enriched in “amide biosynthetic process” and “SRP-dependent cotranslational protein targeting to membrane” in both young and old mice (Fig. S9B). Thus, aging may weaken the role of Th1 and Th17 cells in EAU development.

In response to EAU challenge, the proportion of the naïve B cell subset reduced, and the proportion of other B cell subsets increased in both young and old mice (Fig. S10A–C). We validated the proportional change of plasma cells using flow cytometry. We found higher proportions of plasma cells in old mice both in baseline level and after EAU development (Fig. S10D and S10E). Tfh cells regulate B cell differentiation into plasma cells and memory B cells (Crotty, 2011). The proportion of Tfh cells was higher in ON mice compared to YN mice (Fig. 2C), which increased after EAU development in both young and old mice but in different ratios (Fig. S10F and S10G), and consistent with the proportional change of plasma cells. At the functional level, GO analysis showed that the antigen processing and presentation-related pathway was upregulated only in naïve B cells in young mice while the immunoglobulin production pathway was upregulated only in plasma cells in old mice after EAU development (Fig. S10H and S10I; Tables S3T–W and S4T–W). Plasma cells are the main antibody-producing cells (Lam and Bhattacharya, 2018). The above results thus suggested that plasma cells from old mice had enhanced immunoglobin production capacity, consistent with previous reports of higher titers of autoantibodies in both older people and aged mice (Bovbjerg et al., 1991; Aprahamian et al., 2008). However, these increased serum levels of autoantibodies in old individuals are due to tissue damage and apoptosis (non-specific autoantibody) instead of specific pathologies (specific autoantibody) (Stacy et al., 2002; Aprahamian et al., 2008; Larbi et al., 2008). These increased non-specific autoantibodies including rheumatoid factor and antinuclear antibodies are not necessarily related to diseases (Moulias et al., 1984; Ruffatti et al., 1990). To identify how aging impacted the function of B cells to produce autoantibodies targeting disease-specific autoantigens, we then measured the level of serum immunoglobulins G (IgG) specific to IRBP_1–20_ (anti-IRBP_1–20_ antibody) using ELISA. IRBP_1–20_ is the classical autoantigen that can induce the immune system to attack the retina and accounts for EAU induction (Avichezer et al., 2003). The anti-IRBP_1–20_ IgG was undetectable in YN and ON mice and was markedly lower in OE mice compared to YE mice (Fig. S10J). We also quantified the level of total serum IgG, which was higher in ON mice than in YN mice; whereas after EAU development, YE mice showed higher levels of total IgG than OE mice (Fig. S10K). Thus, aging might lead to enhanced non-specific antibody production of B cells but impaired their capacity to produce specific autoantibodies during autoimmune conditions. Retinal autoantibodies have been proven to augment the pathogenic function of autoreactive T cells in EAU development during adoptive transfer experiments (Pennesi *et al.*, 2003). Thus, the impaired ability of B cells to generate specific autoantibodies may also contribute to the milder EAU symptoms in old mice.

Collectively, our above results delineated alterations in T cell and B cell subsets of CDLNs in response to EAU challenge in both young and old mice. Aging leads to refrained expansion of several CD4^+^ T cell subsets while reserving the proportion dynamics of B cells in response to EAU challenge. In addition, aging reduces specific autoantibody production of B cells during EAU.

### EAU challenge induces divergent cell-cell interaction changes between young and old mice

**Figure 5:**
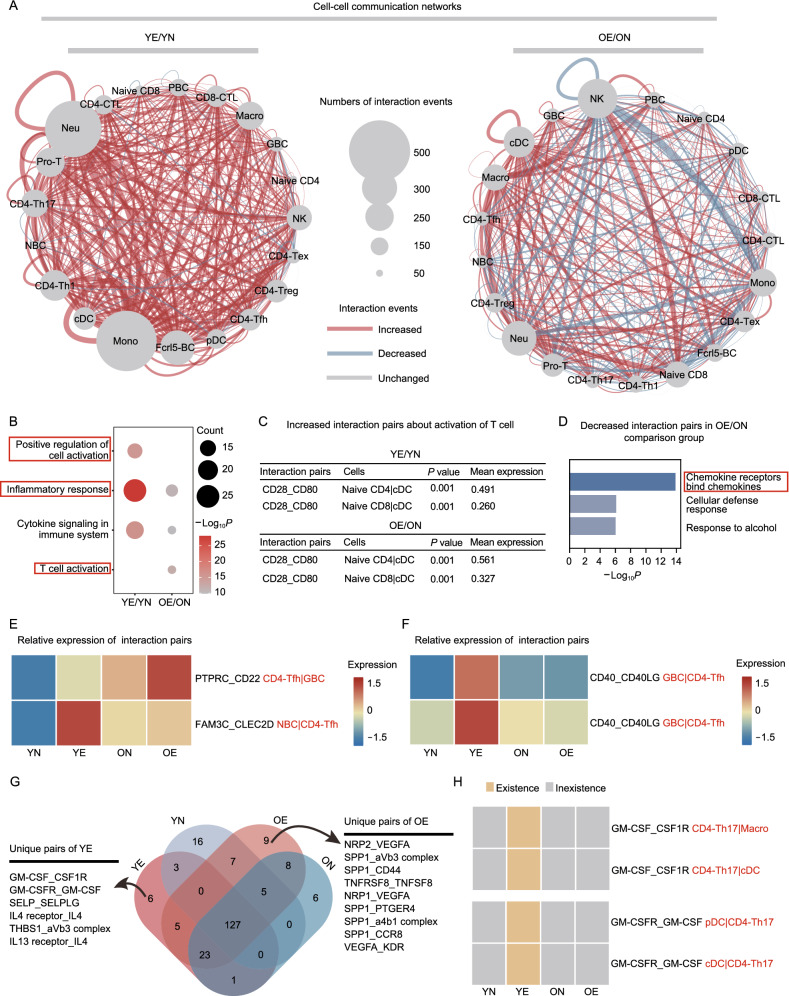
EAU challenge induces divergent cell-cell interaction changes between young and old mice. (A) Network plots showing the changes in ligand-receptor interaction events between different cell types in the YE/YN and OE/ON comparison groups. Cell-cell communication is indicated by the connected line. The thickness of the lines is positively correlated with the number of ligand-receptor interaction events. (B) Functional enrichment analysis showing the GO terms and pathways involving increased ligand-receptor interactions in YE/YN and OE/ON comparison groups. (C) Increased ligand-receptor interactions about T cell activation in YE/YN and OE/ON comparison groups. (D) Functional enrichment analysis showing the GO terms and pathways involving decreased ligand-receptor interactions in OE/ON comparison group. (E) Heatmap of the relative expression in interaction pairs: PTPRC_CD22 between CD4-Tfh and germinal B cells, and FAM3C_CLEC2D between naïve B and CD4-Tfh cells. (F) Heatmap of the relative expression in interaction pairs: CD40_CD40LG between germinal B and CD4-Tfh cells, and between naïve B and CD4-Tfh cells. (G) Venn diagrams showing the numbers of interaction pairs across four groups. The unique interaction pairs of YE are labeled in the diagrams. (H) Expression distribution of GM-CSF and its receptors across the four groups

We also explored the cell-cell interaction among T cells, B cells, and other immune cells of the mice with EAU (Table S5). After EAU development, interaction events were evidently increased among cell types in young mice but were not consistent in old mice (Fig. 5A). The increased interaction events after EAU development were mainly enriched in inflammatory and immune cell activation pathways in both young and old mice, but more evident in young mice (Fig. 5B). In addition, the CD28-CD80 interaction related to T cell activation was increased in both young and old mice after EAU development (Fig. 5C). In old mice, the decreased interaction events after EAU development were enriched in chemokine-related pathway (Fig. 5D), whereas the decreased interaction events were rarely presented in young mice. Additionally, PTPRC-CD22 and FAM3C-CLEC2D interactions related to B cell activation (Llibre et al., 2016; Alborzian Deh Sheikh et al., 2021) were enhanced between B cells and Tfh cells in both young and old mice (Fig. 5E; Table S5) but the CD40-CD40LG interaction, another B cell activation pair, was enhanced only in young mice during EAU (Fig. 5F; Table S5). We further compared the interactions among four groups of mice and identified 6 and 9 pairs of ligand-receptors uniquely increased for YE mice and OE mice, respectively (Fig. 5G). Notably, among these unique pairs, GM-CSF and its receptors have been reported to be closely related to CNS autoimmunity (Lotfi et al., 2019; Chong et al., 2020). We found that the interaction of GM-CSF and its receptors mainly occurred between Th17 cells and APCs (Fig. 5H). These results indicated the interaction of GM-CSF and its receptors between Th17 cells and APCs might contribute to distinct EAU symptoms between young and old mice.

### Aging reduces Th17 pathogenicity by weakening the GM-CSF/IL-23/IL-23R positive feedback loop

**Figure 6:**
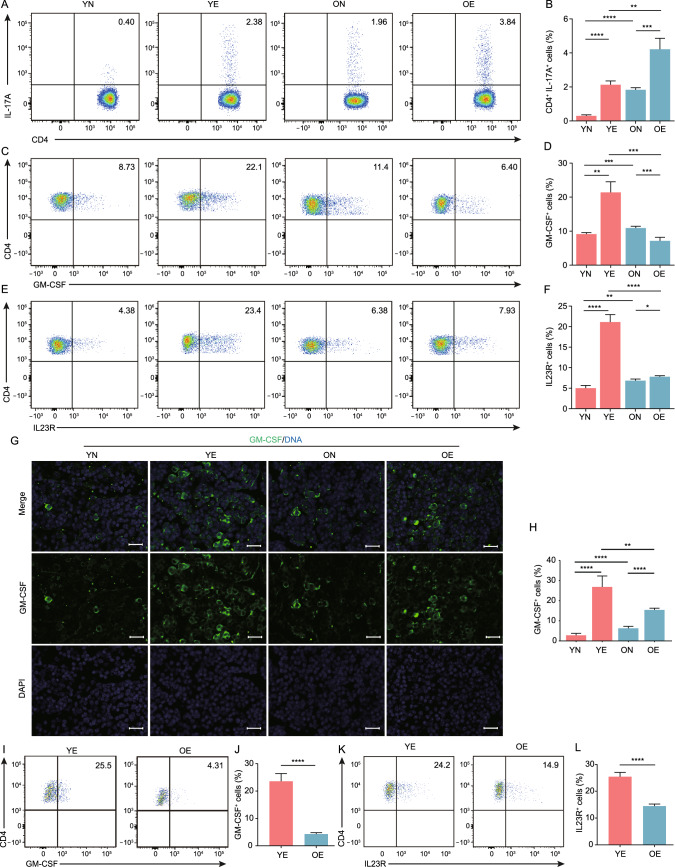
Aging weakened Th17 pathogenicity. (A and B) Representative flow charts (A) and quantification (B) of the proportion of Th17 cells from CDLNs of the four groups. Each group contains six mice. The values represent the mean ± SD from three independent experiments. Significance was determined using two-way ANOVA. **P < 0.01, ***P < 0.001, ****P < 0.0001. (C–F) The proportion of Th17 cells from CDLNs of the four groups expressing GM-CSF (C and D) or IL23R (E and F) was measured by flow cytometry (C and E) and quantified (D and F). Each group contains six mice. The values represent the mean ± SD from three independent experiments. Significance was determined using two-way ANOVA. *P < 0.05, **P < 0.01, ***P < 0.001, ****P < 0.0001. (G and H) Representative images of immunostaining of cross-sections of CDLNs of the four groups for GM-CSF (green) and nuclei (4’,6-diamidino-2-phenylindole (DAPI)-staining; blue) (G) and quantification of GM-CSF^+^ cells (H). Each group contains six mice. The values represent the mean ± SD. Significance was determined using two-way ANOVA. **P < 0.01, ****P < 0.0001. Scale bars, 20 mm. (I–L) The proportion of Th17 cells from retina of YE and OE expressing GM-CSF (I and J) or IL-23R (K and L) was measured by flow cytometry (I and K) and quantified (J and L). Each group contains six mice. The values represent the mean ± SD from three independent experiments. Significance was determined using unpaired student’s *t* test. ****P < 0.0001

Th17 cells are essential in the pathogenicity of AU, and adoptive transfer of Th17 can induce EAU development (Luger et al., 2008). To deeply identify aging-associated changes in Th17 cells’ function during EAU, we analyzed DEGs of Th17 cells across four groups of mice. The expression of *Csf2* and *IL23r* in Th17 cells was higher in young mice compared to old mice after EAU development (Fig. S11A). GM-CSF, encoded by *Csf2*, is dominantly secreted by Th17 cells, which is induced by IL-23 from APCs via IL-23R in Th17 cells. GM-CSF is a hallmark that distinguishes pathogenic Th17 cells from non-pathogenic Th17 cells (Lee et al., 2012; Yasuda et al., 2019), and Th17 cells deficient of GM-CSF lose their pathogenic ability (El-Behi et al., 2011a). Thus, we evaluated GM-CSF and IL-23R expression in Th17 cells by flow cytometry and found that aging increased Th17 cell proportion but reduced their expression of IL-23R and GM-CSF (Figs. 6A–F and S11B). In addition, aging reduced the number of GM-CSF-expressing cells in CDLNs (Fig. 6G and 6H). Correspondingly, the retina-infiltrating T cells in OE mice also exhibited decreased expression of GM-CSF and IL-23R compared to those in YE mice (Fig. 6I–L). These results collectively reflected impaired Th17 pathogenicity led by aging during EAU.

**Figure 7:**
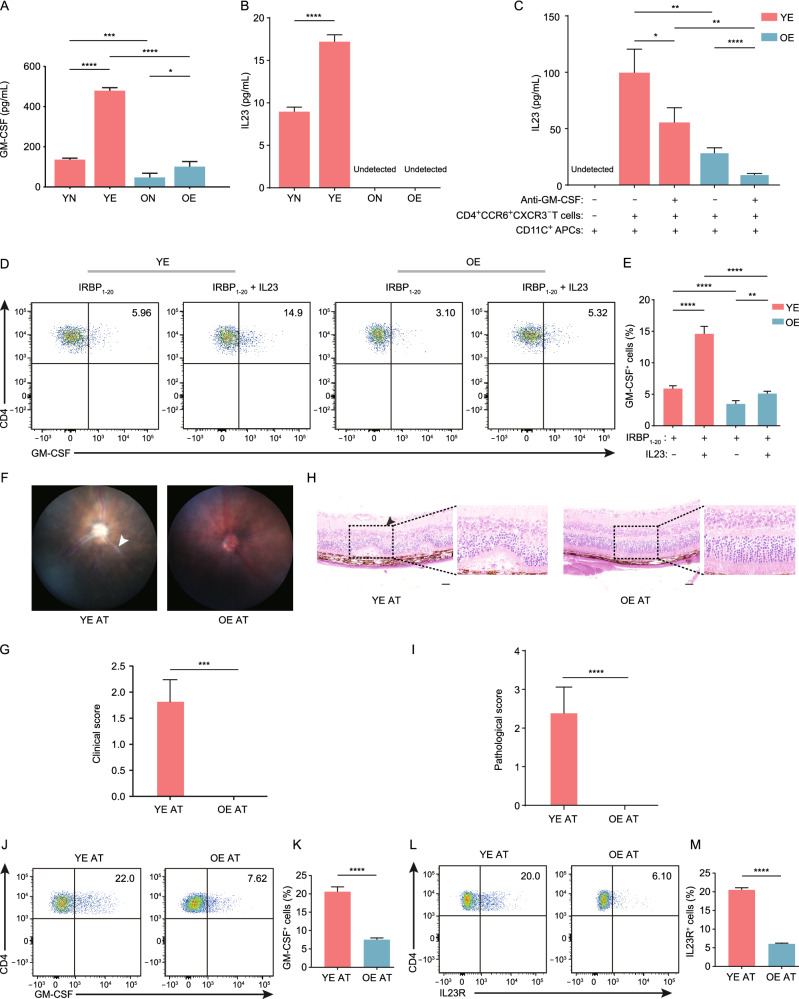
Aging weakened the GM-CSF/IL-23/IL-23R positive feedback loop. (A and B) Serum levels of GM-CSF (A) or IL-23 (B) of the four groups. Each group contains six mice. The values represent the mean ± SD. Significance was determined using two-way ANOVA. *P < 0.05, ***P < 0.001, ****P < 0.0001. (C) CD11C^+^ APCs isolated from YE mice were cocultured with CD4^+^CCR6^+^CXCR3^−^ T cells (Th17 cells) isolated from YE mice or OE mice with or without anti-GM-CSF antibody for 72 h. IL-23 level in the culture supernatant was measured by ELISA. The values represent the mean ± SD from six independent experiments. Significance was determined using two-way ANOVA. *P < 0.05, **P < 0.01, ***P < 0.001. (D and E) CDLN cells from YE and OE mice were collected after immunization on day 14 and cultured with IRBP_1–20_ alone or with IRBP_1–20_ plus IL–23. The proportion of GM-CSF-expressing Th17 cells was measured by flow cytometry (D) and quantified (E). The values represent the mean ± SD from six independent experiments. Significance was determined using two-way ANOVA. **P < 0.01, ****P < 0.0001. (F and G) The representative fundus images (F) and clinical scores (G) after induction by CD4^+^CCR6^+^CXCR3^−^ T cells (Th17 cells) from YE (YE-AT) or OE (OE-AT) groups. Each group contains six mice. Significance was determined using unpaired student’s *t*-test. ***P < 0.001. White arrowheads indicate inflammatory exudation and vascular deformation. (H and I) The representative HE staining images (H) and pathological scores (I) after induction by CD4^+^CCR6^+^CXCR3^−^ T cells (Th17 cells) from YE (YE-AT) or OE (OE-AT) groups. Black arrowheads indicate magnified area with infiltration of inflammatory cells and retinal folding. Scale bars, 20 mm. Each group contains six mice. Significance was determined using unpaired student’s *t*-test. ****P < 0.0001. (J–M) The proportion frequency of Th17 cells from CDLN of YE (YE-AT) or OE (OE-AT) groups expressing GM-CSF (J and K) or IL-23R (L and M) was measured by flow cytometry (J and L) and quantified (K and M). Each group contains six mice. The values represent the mean ± SD from three independent experiments. Significance was determined using unpaired student’s *t*-test. ****P < 0.0001

Interactions of GM-CSF and its receptors between Th17 cells and APCs only occurred in young mice but not in old mice after EAU development (Fig. 5H). GM-CSF secreted by Th17 cells can augment the secretion of IL-23 from APCs and vice versa, thus constituting a positive feedback loop to maintain Th17 pathogenicity via IL23R expressed in Th17 cells (El-Behi et al., 2011a). The reduced GM-CSF and IL23R expression in Th17 cells may therefore account for abolished interactions between Th17 cells and APCs and may influence IL-23 secretion by APCs. Indeed, we detected decreased serum levels of GM-CSF in old mice by ELISA (Fig. 7A), which was consistent with low GM-CSF levels in elder people (Kim et al., 2011). Additionally, the concentration of IL-23 in serum from old mice was also reduced (Fig. 7B).

To explore the above hypothesis, we isolated CD11C^+^ APCs from YE mice and cocultured them with CD4^+^CCR6^+^CXCR3^−^ T cells (Th17 cells) (Zhao et al., 2020) from CDLNs of YE or OE mice. Coculture promoted IL-23 secretion by APCs, and Th17 cells from YE mice have a more potent promoting function on APCs than those from OE mice (Fig. 7C). Additionally, IL-23 secretion was decreased after GM-CSF neutralization (Fig. 7C). This result demonstrated that GM-CSF secreted by Th17 cells was involved in the induced expression of IL-23 in APCs. Moreover, low GM-CSF secretion by Th17 cells and consequently low IL-23 secretion by APCs may in turn, further impair Th17’s pathogenicity due to the compromised IL23-IL23R interaction between APC and Th17 cells. To confirm this in IRBP_1–20_-specific response, IRBP_1–20_ was used as a stimulator. We collected CDLN cells from YE and OE mice, cultured these cells with IRBP_1–20_ alone or with IRBP_1–20_ plus IL-23, and measured the GM-CSF secretion by Th17 cells. IL-23 enhanced the secretion of GM-CSF by Th17 cells from YE mice more than that by Th17 cells from OE mice, consistent with aging-induced downregulation of IL-23R in OE mice (Fig. 7D and 7E). Thus, our study demonstrated that aging weakened Th17 pathogenicity, which was to some extent, attributed to the downregulated expression of IL-23R. We further conducted an adoptive transfer experiment. Th17 cells isolated from CDLNs of YE mice induced uveitis in mice whereas these cells isolated from OE mice failed to induced EAU in naïve mice (Fig. 7F–I). Correspondingly, a significantly less amount of GM-CSF^+^ and IL-23R^+^ Th17 cells were detected in mice that received Th17 cells from OE mice (Fig. 7J–M). This result further confirmed the impaired Th17 cell pathogenicity induced by aging. These results collectively indicated that aging weakened Th17 pathogenicity by weakening the GM-CSF/IL-23/IL-23R positive feedback loop (Fig. S11C).

## DISCUSSION

Here, using scRNA-seq, we are the first to map the immune cell atlas of aging LNs. Combining with flow cytometry, we deeply investigate the impact of aging on EAU. The result that old mice developed milder EAU can be attributed to the extensive alterations induced by aging in immune cell response to EAU challenge. Importantly, within this process, our study unveils reduced pathogenicity of Th17 cells from aging mice, reflected as low GM-CSF secretion by Th17 cells resulting from aging-induced downregulation of IL-23R in these cells. These defective Th17 cells contribute to low IL-23 secretion of APCs, which may further impair APCs’ promoting effects on pathogenic Th17 cells.

The impact of aging on immune system has tissue specificity and stage specificity (Consortium., 2020). Although alterations of the immune system during aging have been extensively studied in human peripheral blood and mouse spleen (Elyahu et al., 2019; Zheng et al., 2020), a detailed immune cell atlas in aged lymph nodes, the essential constituents of the immune system, was still missing before our study, let alone a detailed study of cellular alterations in aged CDLNs and associated autoimmune diseases. Here, we for the first time delineate the immune cell atlas of CDLNs of both young and old mice with or without EAU through a combination of scRNA-seq and flow cytometry. It is widely known that aging is commonly associated with the reduction of naïve cells and the expansion of antigen-experienced subsets (Nikolich-Žugich, 2018). We also detected a decrease of naïve subsets and an increase of effector subsets in T cells of aged CDLNs, which might be associated with enhanced differentiation of naïve subsets in old mice compared to young mice as indicated by our GO and pseudotime analyses. In the functional perspective, consistent with previous reports that aging of the immune system was usually accompanied by chronic, low-grade inflammation (Franceschi et al., 2017), DEG and GO analyses of total CDLN immune cells also indicated an inflammatory state of aged CDLNs. Additionally, GO analysis of T cells showed multiple effector states (a mix of inflammatory, cytotoxic, and regulatory phenotypes) of T cells from aging mice, similar to the results of the previous report (Elyahu et al., 2019). Notably, we also observed impaired Th17 cells, which have not gained enough attention before our study, and now we have accentuated its importance in autoimmune responses, especially with respect to age. Regarding B cells, preserved and even enhanced immunoglobin production capacity of plasma cells is indicated by our study. With regard to changes of innate immune cells during aging, varied conclusions had been presented (Shaw et al., 2010; Solana et al., 2012). The proportions of DC, macrophages, NK cells, and neutrophils were reported to be maintained or even increased during aging (Tesar et al., 2006; Shaw et al., 2010; Solana et al., 2012). Our study provided additional evidence showing an increased proportion of innate immune cells except for NK cells during aging (Fig. 1D). At the functional level, GO analysis indicated decreased antigen-presentation capacity of DC and reduced cytotoxicity of NK cells as previously reported (Fig. 1G) (Uyemura et al., 2002; Solana et al., 2006). In addition, it has been proposed that activation of the innate immune system is one of the features of chronic inflammations in the elderly (Franceschi et al., 2018). Consistently, GO analysis of DEGs for innate immune cells provides such evidence, such as the activation of the neutrophils and the shift towards pro-inflammatory phenotype of monocytes and macrophages in the old mice. In summary, aging induces complicated changes in the immune cellular constitutes of CDLNs. These changes are generally consistent with changes in the general immune system induced by aging that have been widely studied (Solana et al., 2012; Nikolich-Žugich, 2018).

The extensive alterations of immune cells indicate an altered autoimmune process in old individuals. However, little attention has been paid to the relationship between aging and autoimmunity before. Our study deliberately compared the autoimmune response to EAU challenge between young and old mice, which may provide references to a wider range of autoimmune diseases. In our study, old mice show much milder EAU symptoms compared to young mice. In addition to being the main orchestrators of adaptive immunity, T cells and B cells significantly contribute to autoimmune diseases (Khan and Ghazanfar, 2018; Meffre and O'Connor, 2019). In AU, T cells are the initiators and play essential roles in its pathogenesis (Weaver et al., 2006; Dittel, 2008), whereas the role of B cells is still uncertain (Smith et al., 2016; Zhu et al., 2021). Our GO analyses showed that T and B cells from young and old mice exhibited distinct upregulated pathways, suggesting their important role in divergent EAU symptoms between young and old mice. The IL-17 signaling was enhanced in young mice but not in old mice after EAU development within both total immune cells and the T cell compartment. This result is in line with the impaired function of Th17 cells in old mice and implicates its role in the aging-induced divergent autoimmune response to EAU challenge. About B cell subsets, although our scRNA-seq data indicated increased immunoglobin production by plasma cells in old mice, ELISA detected decreased IRBP_1–20_-specific autoantibody production by plasma B cells in old mice. This divergence may result from age-associated defects in the induction of key molecules responsible for class switching and somatic hypermutation (Frasca et al., 2004). In addition, age-related defects in CD4^+^ T cell cognate helper function also contribute to lower levels of antigen-specific IgG (Eaton et al., 2004). The function of retinal autoantibodies secreted by B cells in EAU has been reported. Although the serum of EAU mice (containing autoantibodies targeting the retina) alone failed to transfer diseases to recipients, transferring autoreactive T cells with that serum can exacerbate EAU symptoms compared to transferring T cells alone, which indicated that retinal autoantibodies may play an assisting role in EAU development (Pennesi et al., 2003). Thus, aging-related decline of antigen-specific autoantibodies in plasma cells may contribute to the milder EAU symptoms. As for innate immune cells, DC from old mice show impaired upregulation in antigen-presentation pathways, whereas no difference was evident in the other innate immune cell types between young and old mice in response to EAU challenge. Overall, aging induces altered immune cell response to EAU challenge, which may counteract EAU challenge in old mice.

Th17 cells are critical pathogenic components of AU and EAU (Amadi-Obi et al., 2007; Wilson et al., 2007). The pathogenicity of Th17 cells can be modulated by environmental factors, such as a high-salt diet (Kleinewietfeld et al., 2013) or environmental toxins (Veldhoen et al., 2008). However, few studies have reported altered pathogenesis of Th17 cells as a consequence of aging and the underlying mechanisms. As discussed above, the function of Th17 cells in EAU development might be weaker in old mice compared to that in young mice. Among the unique ligand-receptor interactions of YE mice that may account for divergent EAU processes between young and old mice, the interaction between GM-CSF (encoded by *Csf2*) and its receptors has a close relationship to CNS autoimmunity (Ifergan et al., 2017). Expression of GM-CSF is a mark of pathogenic Th17 cells (Codarri et al., 2011; El-Behi et al., 2011b). Flow cytometry showed that Th17 cells of old mice lost their pathogenicity, which was reflected as reduced GM-CSF secretion, during EAU. In addition, Th17 cells defective in GM-CSF expression reduced the IL-23 secretion by cocultured APCs. This effect further impaired the promoting effect of APCs on pathogenic Th17 cells (El-Behi et al., 2011a). GM-CSF is a downstream molecule of IL-23R and the stimulation of IL-23 can induce GM-CSF secretion (El-Behi et al., 2011b; Komuczki et al., 2019). The necessity of IL-23 in Th17 pathogenicity has been demonstrated (El-Behi et al., 2011b; Komuczki et al., 2019). Our scRNA-seq data showed that the expression of *IL-23R* was lower in old mice compared to that in young mice after EAU development, which was confirmed by flow cytometry. Our *in vitro* study further supported the correlation of lower IL-23R levels with reduced GM-CSF secretion by Th17 cells from old mice. Taken together, our study is the first to demonstrate that aging weakened Th17 pathogenicity by dampening the GM-CSF/IL-23/IL-23R positive feedback loop.

As for the stage specific contribution of aging to uveitis, this disease is less common in children, with enhanced symptoms and increased incidence with age in people of working age (20 to 50 years of age), but much less severe in the elderly people (>65-year-old) (Papotto et al., 2014; Tsirouki et al., 2016). However, the proportion of Th17 cells, the main pathogenic cell type in uveitis, gradually and continuously increases during aging (ranging from newborn to 60–79-year-old) (Botafogo et al., 2020). Wang et al. demonstrated that, in middle-aged mice (10-month-old), the accumulated DNA and the regulatory subunit of the DNA-dependent protein kinase augment the activation of CD4^+^ T cells and aging-related auto-inflammation (Wang et al., 2021). This study explained the mechanism of higher incidence of autoimmune uveitis in young adults. However, in the elderly people, it waits to be explained for the lower incidence of autoimmune uveitis and the seemingly contradicting, higher proportion of Th17 cells. And our study filled this gap. We demonstrated that dampening the GM-CSF/IL-23/IL-23R positive feedback loop is key to the weakened pathogenicity of Th17 cells in the old individuals, even though the proportion of Th17 cells is higher in old mice than young mice. Thus, the results of the above study in middle-aged mice and our study in old mice reflect the divergent characteristics of autoimmune inflammation in people of different age groups and actually complement each other.

In summary, our study delineates an immune cell atlas of aging CDLNs and explores the impact of aging on autoimmune response based on EAU model. We demonstrate enormous changes in immune cells in response to EAU challenges between young and old mice that may account for milder EAU symptoms in old mice. Importantly, we are the first to report that aging weakens Th17 pathogenic function and its underlying mechanisms. Our study provides novel insights into the interplay between aging and autoimmunity and serves as a reference for a large community of scientists interested in this subject.

## MATERIALS AND METHODS

### Mice

Young (2 months old) and old (20 months old) C57BL/6J mice were purchased from SPF Biotechnology Co., Ltd. (Beijing, China). The animal experiments were approved by the Institutional Animal Care Committee (Zhongshan Ophthalmic Center, Sun Yat-Sen University).

### EAU model induction and EAU clinical score

For active induction of EAU, mice were injected subcutaneously with an emulsion consisted of 2 mg/mL of retinal antigen interphotoreceptor retinoid-binding protein 1–20 (IRBP_1–20_) (GPTHLFQPSLVLDMAKVLLD, GiL Biochem, Shanghai, China) and complete Freund’s adjuvant (BD Difco, San Jose, CA, USA) containing 2.5 mg of Mycobacterium tuberculosis strain H37Ra (BD Difco, San Jose, CA, USA) in a 1:1 volume ratio. Additionally, 0.25 µg pertussis toxin (PTX) (List Biological Laboratories, Campbell, California, USA) dissolved in PBS was injected on the same day and 2 days after immunization (Chan et al., 1990; Agarwal et al., 2012; Chen and Caspi, 2019b).

Funduscopic examination of EAU progress with the Micron IV fundus camera (Phoenix Co., Campbell, CA, USA) was performed and the clinical findings were graded from 0 to 4 based on observable infiltration and vasculitis in the retina (Details in Table S6). The clinical score was assessed in a blinded manner.

### Flow cytometric analysis

After staining with live/dead dye (Thermo Fisher Scientific, Waltham, MA, USA) for 10 min at 4 °C and washing once with PBS, the cells were stained with antibodies detecting surface markers: CD4 (GK1.5, catalog 100434, BioLegend), IL-23R (12B2B64, catalog 150907, BioLegend), CXCR3 (CXCR3-173, catalog 126506, BioLegend), CCR6 (29-IL17, catalog 129819, BioLegend) and analyzed via flow cytometry (BD LSRFortessa). For intracellular cytokine staining, the cells were stimulated with 5 ng/mL of phorbol myristate acetate (Sigma), 1 μg/mL brefeldin A (Sigma), and 500 ng/mL ionomycin (Sigma) at 37 °C for 4 h. After fixation and permeabilization, harvested cells were stained with antibodies detecting: IL-17A (TC11-18H10.1, catalog 506930, BioLegend), GM-CSF (MP1-22E9, catalog 2131415, Invitrogen). Finally, the cells were measured by flow cytometry. The results were evaluated with FlowJo software (version 10.0.7, Tree Star, Ashland, OR, USA).

### Treatment of Draining Lymph node Cells

CDLNs cells were stimulated by IRBP_1–20_ (20 ng/mL) with or without IL-23 (20 ng/mL, PeproTech, Rocky Hill, NJ, USA) at 37 °C for 72 h in a humidified incubator with 5% CO_2_. The cells were analyzed by flow cytometry.

### Co-culture of CD4^+^CCR6^+^CXCR3^−^ T cells with CD11C^+^ APCs

CD4^+^CCR6^+^CXCR3^−^ T cells (Th17 cells) were isolated by cell sorting and CD11C^+^ APCs were isolated by CD11C positive selection kit (STEMCELL, Vancouver, Canada). Then, sorted CD4^+^CCR6^+^CXCR3^−^ T cells and CD11C^+^ APCs were co-cultured at a ratio of 2:1 with or without anti-GM-CSF (10  g/mL, R&D Systems) for 24 h. The supernatants were used to measure IL-23 by ELISA kit (Invitrogen, Carlsbad, CA, USA).

### EAU induction by the adoptive transfer experiment

CD4^+^CCR6^+^CXCR3^−^ T cells (Th17 cells) sorted from CDLNs of old and young EAU mice were stimulated with IRBP_1–20_ (20 μg/mL) for 72 h, washed with PBS three times, then injected through the tail vein into young or old C57BL/6j mice (2 × 10^7^ living cells/ mice).

### Pathological examination of eyes

Different groups of mice were euthanized humanely, their eyes were extracted and placed in 10% neutral-buffered formalin for at least 24 h. The fixed eyes were dehydrated in alcohol and embedded in paraffin. Finally, paraffin blocks were sectioned at a thickness of 4 μm. The sections were photographed by a microscope system after staining with hematoxylin and eosin. For each eye, four sections from four different layers were graded. The pathological scores were graded with a score between 0 to 4 as previously described (Chen and Caspi, 2019a). Scoring criteria in details was already in the Table S6.

### IRBP antibody assay

Serum levels of mice antibody against IRBP_1–20_ (IgG) from different groups of mice after immunization were quantified by ELISA. Briefly, the wells were coated with 1 µg/mL IRBP_1–20_ at 4 °C overnight then blocked with 3% bovine serum albumin in PBS at 4 °C for 2 h. Diluted serum samples at 1:100 in 3% bovine serum albumin (BSA) were added to the wells. After incubation at 37 °C for 1.5 h to allow the antibody to bind the antigen, the sera were removed, and the wells were washed. Bound antibody was detected with goat anti-mouse IgG diluted at 1:2,000 for 1.5 h (Southern Biotechnology, Birmingham, AL, USA). Absorbance was read at 450 nm and mean optical density (OD ± SEM) was calculated for each group after adding stop solution.

### Immunofluorescence staining

CDLNs were removed and fixed with 4% paraformaldehyde overnight at 4 °C, dehydrated using sucrose with different concentration gradients (10%, overnight; 20%, 6–8 h; 30%, overnight) and embedded in Tissue-Tek O.C.T Compound (Sakura Finetek USA, Inc). Sagittal sections (4 µm) were cut and blocked with 3% bovine serum albumin in PBS for 30 min at 23 °C ± 2 °C. The sections were incubated with anti-GM-CSF antibodies (1:100, MP1-22E9, catalog 2131415, Invitrogen) overnight at 4 °C and further incubated with the corresponding fluorochrome-conjugated secondary antibody (1:1,000, Cell Signaling Technology, Danvers, MA, USA) for 50 min. After counterstaining with DAPI (catalog ab104139, Abcam), the sections were examined with a fluorescence microscope (Nikon, Tokyo, Japan). The number of immunoreactive cells or percentage of immunoreactive area (20× objective) was analyzed via ImageJ software (v1.46r, NIH, Bethesda, MD, USA).

### Enzyme-linked immunosorbent assay (ELISA)

Mouse sera or supernatants form cell coculture medium were analyzed with the following ELISA kits according to the manufacturer’s instructions: IL-23 Mouse ELISA Kit (BMS6017, Thermo Fisher Scientific) and GM-CSF Mouse ELISA Kit (BMS612, Thermo Fisher Scientific).

### Single-cell collection

CDLNs were transferred to DMEM (Thermo Fisher Scientific) containing 2% FBS (Thermo Fisher Scientific), Pen/Strep antibiotics (Thermo Fisher Scientific), 3 mg/mL Collagenase IV (Sigma-Aldrich) and 40 mg/mL DNase I (Sigma-Aldrich), and incubated at 37 °C for 15 min. Digested cells were collected and filtered through a 70 um cell strainer. The single cell suspension was generated at 1 × 10^7^ cells/mL (viability ≥ 85%) as determined using the Countess® II Automated Cell Counter.

### scRNA-seq library preparation

scRNA-seq libraries were generated utilizing the Chromium Single Cell 5’ Library and Gel Bead Kit (10× Genomics, 120237) according to the manufacturer’s instruction with some modifications. In detail, after washing with 0.04% BSA buffer (0.02 g BSA dissolved in 50 mL deionized PBS), cells were captured in droplets. Then, reverse transcription, emulsion breaking, barcoded-cDNA purification with Dynabeads, and PCR amplification were conducted step by step. The amplified cDNA was then used for 5’ gene expression library construction. Specifically, fragmenting and end-repair, double-size selection with SPRIselect beads, and sequencing were conducted on 50 ng of amplified cDNA using NovaSeq platform (Illumina NovaSeq6000) to yield 150 bp paired-end reads.

### Quality control

Raw data (Raw reads) of fastq files were assembled from the Raw BCL files using Illumina’s bcl2fastq converter. For primary quality control with FastQC software (S Andrews, 2010), the following parameters were assessed, (1) contained N was no more than 3; (2) the proportion of base with quality value below 5 was no more than 20%; (3) adapter sequence was removed. All the downstream analyses were based on the cleaned data with high quality.

### Analysis of scRNA-seq data

The command “cellranger count” in CellRanger Software Suite (version 3.1.0) was used to demultiplex and barcode the sequences from NovaSeq system. Calculation of the single-cell expression matrix was performed via the Seurat software (version 2.3.4) for filtering, data normalization, dimensionality reduction, clustering, and differentially expressed gene analysis (Butler et al., 2018).

### Quality control

Before proceeding further, cells with a number of genes <300 or >4,000, or with a ratio of mitochondrial genes >15% were excluded. Most cells with high expression of *Hbb-a1* and *Hbb-bs* that were recognized as red blood cells were filtered. The batch effect across different samples was removed with the Harmony package (version 1.0) (Korsunsky et al., 2019).

### Dimensionality reduction and clustering analysis

For analysis of scRNA-seq data with Seurat, the data were normalized via “NormalizeData’’ function, followed by PCA on top 2,000 variable genes via ‘‘FindVariableGenes’’ function with the default parameters. ‘‘FindClusters’’ function was used to cluster cells and ‘‘RunUMAP’’ function was used to visualize with a 2-dimensional UMAP algorithm. In addition, ‘‘FindAllMarkers’’ function with default parameters was used to generate marker genes of different clusters.

### Cell type composition variation analysis

The number of cells from different cell types across YN, YE, ON, and OE mice were divided by the total number of cells in the same group to generate the cell type ratio. The Log_2_fold-change (Log_2_FC) in YE/YN, OE/ON, OE/YE comparison groups were used to recognize the alteration of cell types during aging and EAU (|Log_2_FC| > 0.5).

### DEG analysis

DEG analysis from different kinds of cells between different groups (ON/YN, YE/YN, OE/ON) was performed by ‘‘FindMarkers’’ function (adjusted *P* value < 0.05, |LogFC| > 0.25). Before DEG analysis, we excluded the cell types that were missing or had fewer than three cells in the comparison groups.

### Gene Ontology enrichment analysis

The Metascape webtool (www.metascape.org) (Zhou et al., 2019) was used to perform DEGs gene ontology (GO) and pathway enrichment analysis. Among the top 50 enriched GO terms or pathways across different kinds of cells, 5–10 GO terms or pathways that were associated with aging or EAU were visualized with ggplot2 (Ginestet, 2011).

### Cell-cell communication

The intercellular communication was performed with CellPhoneDB (version 1.1.0) (Vento-Tormo et al., 2018). We selected and analyzed the ligand-receptor pairs that existed in more than 10% of a given cell type. We compared the mean of expression from ligand-receptor pairs in different cell types then selected pairs with *P* < 0.05 for further computerization of intercellular communication.

### Cell–cell signaling pathways

Cell–cell signaling pathways were analyzed using the CellChat package (version 1.1.0) (Jin et al., 2021) to predicts major signaling inputs and outputs for cells and how those cells and signals coordinate for functions by network analysis and pattern recognition approaches.

### Transcriptional factor (TF)

According to the workflow (http://scenic.aertslab.org/), TF-binding motifs were performed with the GENIE3 packages (version 1.6.0) (Huynh-Thu et al., 2010) and the RcisTarget database (version 1.4.0) (Verfaillie et al., 2015) of the SCENIC (version 1.1.2.2) (Aibar et al., 2017). TFs were identified by SCENIC only if they were found to be active in more than 1% of all cells and correlated with at least one other regulon (|r| > 0.3).

### Pseudotime analysis

Pseudotime analysis was performed with the Monocle2 package (Qiu et al., 2017). Gene ordering was performed by using a cutoff of expression in at least 10 cells and a combination of intercluster differential expression and dispersion with a q-value cutoff of <0.1. The structure of the trajectory was plotted in 2-dimensional space using the DDRTree dimensionality reduction algorithm, and the cells were ordered in pseudotime.

### Statistical analysis

GraphPad Prism Software was used for data analysis and presentation. The values are represented as the mean ± SD. Statistical analysis was performed with an unpaired, two-tailed Student’s *t*-test or one-way ANOVA. *P* values above 0.05 were considered as not significant, ns; *, *P* < 0.05; **, *P* < 0.01; ***, *P* < 0.001; and ****, *P* < 0.0001.

### Data availability statement

The single-cell sequencing data have been deposited at Genome Sequence Archive (GSA) with the project number PRJCA006064 and GSA accession number CRA004687.

## ABBREVIATIONS

APCs, antigen-presenting cells; AU, autoimmune uveitis; BC, B cells; BSA, bovine serum albumin; CD4-CTL, CD4^+^ T cells with cytotoxic activity; CD8-CTL, CD8^+^ T cells with cytotoxic activity; CNS, central nervous system; CDLNs, cervical draining lymph nodes; cDC, conventional dendritic cells; DEGs, differentially expressed genes; EAU, experimental autoimmune uveitis; FCGR, Fc-gamma receptor; Fcrl5-BC, Fcrl5-positive B cells; GO, Gene Ontology; GBC, germinal B cells; GC, germinal center; H&E, Hemotoxylin and eosin; IgG, immunoglobulins G; IRBP1-20, interphotoreceptor retinoid-binding protein 1-20; LNs, Lymph nodes; Macro, macrophages; Mono, monocytes; NBC, naïve B cells; naïve CD4, naïve CD4^+^ T cells; naïve CD8, naïve CD8^+^ T cells; NK, natural killer cell; Neu, neutrophils; OE, old mice with EAU; ON, old normal mice; PTX, pertussis toxin; PBC, plasma B cells; pDC, plasmacytoid dendritic cells; Pro-T, proliferative T cells; scRNA-seq, single-cell RNA sequencing; TC, T cells; Tex, exhausted T cells; Tfh, T follicular helper cells; TFs, transcriptional factors; Th1, T helper 1 cells; Th17, T helper 17 cells; Treg, regulatory T cells; YE, young mice with EAU; YN, young normal mice.

## DECLARATIONS

This work was supported by the National Key Research and Development Program of China (2017YFA0105804), the Strategic Priority Research Program of the Chinese Academy of Sciences (XDA16010000), the National Key Research and Development Program of China (2020YFA0804000, 2018YFC2000100, 2017YFA0103304, 2020YFA0803401, 2019YFA0802202), the National Natural Science Foundation of China (Grant Nos. 81921006, 81625009, 91749202, 91949209, 81822018, 82125011, 82122024, 31970597), the Key Research Program of the Chinese Academy of Sciences (KFZD-SW-221), and the 14th Five-year Network Security and Informatization Plan of Chinese Academy of Sciences (WX145XQ07-18). He Li, Lei Zhu, Rong Wang, Lihui Xie, Jie Ren, Shuai Ma, Weiqi Zhang, Xiuxing Liu, Zhaohao Huang, Binyao Chen, Zhaohuai Li, Huyi Feng, Guang-Hui Liu, Si Wang, Jing Qu, and Wenru Su declare that they have no conflict of interest. All procedures followed were in accordance with the ethical standards of the Institutional Animal Care Committee (Zhongshan Ophthalmic Center, Sun Yat-Sen University). He Li, Lei Zhu, Rong Wang, Lihui Xie, Jie Ren, Shuai Ma, Weiqi Zhang, Xiuxing Liu, Zhaohao Huang, Binyao Chen, Zhaohuai Li, Huyi Feng, Guang-Hui Liu, Si Wang, Jing Qu, and Wenru Su declare that they agreed to participate in the project. He Li, Lei Zhu, Rong Wang, Lihui Xie, Jie Ren, Shuai Ma, Weiqi Zhang, Xiuxing Liu, Zhaohao Huang, Binyao Chen, Zhaohuai Li, Huyi Feng, Guang-Hui Liu, Si Wang, Jing Qu, and Wenru Su declare that they agreed to publish the project. The single-cell sequencing data is deposited in the Genome Sequence Archive in BIG Data Center, Beijing Institute of Genomics (BIG, https://bigd.big.ac.cn/gsa/), Chinese Academy of Sciences, with the project number PRJCA006064 and GSA accession number CRA004687.

## Supplementary Information

Below is the link to the electronic supplementary material.

Supplementary file1 (PDF 5610 kb)

Supplementary file2 (XLSX 533 kb)

Supplementary file3 (XLSX 19 kb)

Supplementary file4 (XLSX 365 kb)

Supplementary file5 (XLSX 274 kb)

Supplementary file6 (XLSX 703 kb)

Supplementary file7 (XLSX 10 kb)
