# Supplementary material for: Aging weakens Th17 cell pathogenicity and ameliorates experimental autoimmune uveitis in mice
Source: Protein Cell. 2021 Nov 8;13(6):422–45. doi: 10.1007/s13238-021-00882-3 (PMC9095810; doi:10.1007/s13238-021-00882-3)
Supplement: Supplementary file 1 — Supplementary file1 (PDF 5610 kb) [file 13238_2021_882_MOESM1_ESM.pdf]

Supplementary Legends

Supplementary Fig. S1 Clusters of immune cell subsets, deferentially expressed genes of T cells, and GO analysis of immune cell subsets in ON and YN groups.

A. Heatmap showing scaled expression of discriminative gene sets for immune cell subsets in CDLNs.
B. UMAP plots of canonical markers for immune cell subsets.
C. Representative GO terms and KEGG pathway enriched in downregulated DEGs of total immune cells in ON/YN comparison group.
D. Volcano plot showing up- and down-regulated DEGs in T cells in the ON/YN comparison group. Red and blue plots indicated up- and down-regulated DEG of T cells in ON group compared to YN group, respectively.
E. Representative GO terms and KEGG pathway enriched in downregulated DEGs of immune cell subsets in the ON/YN comparison group.

Supplementary Fig. S2 Re-clustering, and pseudotime trajectory analysis of T cells in the ON/YN comparison group.

A. Heatmap showing scaled expression of discriminative gene sets for T cell subsets in CDLNs.
B. UMAP plots of canonical markers for T cell subsets.
C. Pseudotime trajectory analysis corresponding to the differentiation of effector $CD4^+$ T cells from naïve $CD4^+$ T cells.
D. Expression transition of differentiation-associated genes along the pseudotime.

Supplementary Fig. S3 Pseudotime trajectory analysis of $CD8^+$ T cells, differentially expressed genes and GO analysis of naïve T cells in the YN/ON comparison group.

A. Pseudotime trajectory analysis corresponding to the differentiation of effector $CD8^+$ T cells from naïve $CD8^+$ T cells in YN and ON group, respectively. Cells are colored by pseudotime (top) or cell type (bottom).
B. Pseudotime trajectory analysis corresponding to the differentiation of effector $CD8^+$ T cells from naïve $CD8^+$ T cells in both YN and ON groups. Cells are colored by pseudotime.
C. Percentages of $CD8^+$ T cells along the pseudotime for YN and ON mice.
D. Expression transition of differentiation-associated genes along the pseudotime.
E-F. Volcano plot showing up- and down-regulated DEGs of naïve $CD4^+$ T cells (E) or naïve $CD8^+$ T cells (F) in the ON/YN comparison group. Red and blue plots indicated up- and down-regulated DEG in ON group compared to YN group, respectively.
G. Representative GO terms and KEGG pathway enriched in upregulated DEGs of naïve $CD8^+$ T cells in the ON/YN comparison group.
H-I. Violin plots of expression level of *Il17a* and *Il1r1* in T subsets from YN and ON groups.

Supplementary Fig. S4 GO analysis of T cell subsets in the ON/YN comparison group.

A-B. Representative GO terms and KEGG pathway enriched in upregulated (A) or downregulated (B) DEGs of T cell subsets in the ON/YN comparison group.

Supplementary Fig. S5 Re-clustering, and Pseudotime trajectory analysis of B cells in the ON/YN comparison group.

A. UMAP plot showing clusters of B cell subsets.

B. Heatmap showing scaled expression of discriminative gene sets for B cell subsets.
C. UMAP plots of canonical markers for B cell subsets.
D. Bar chart of the relative proportion of B cell subsets in the ON/YN comparison group derived from scRNA-seq data.
E. Pseudotime trajectory analysis corresponding to the differentiation of effector B cells from naïve B cells in YN and ON groups, respectively. Cells are colored by pseudotime, cell type, as indicated, from the top to the bottom panels.
F. Pseudotime trajectory analysis corresponding to the differentiation of effector B cells from naïve B cells in both YN and ON groups. Cells are colored by pseudotime.
G. Percentages of B cells along the pseudotime for YN and ON mice.
H. Expression transition of differentiation-associated genes along the pseudotime.

Supplementary Fig. S6 GO analysis of B cells subsets and cell-cell interactions of immune cell subsets in the ON/YN comparison group.
A-B. Representative GO terms and KEGG pathway enriched in upregulated (A) or downregulated (B) DEGs of B cell subsets in the ON/YN comparison group.
C. Heatmap of relative expression of activation-related interaction pairs from T cells and B cells in the ON and YN groups.
D. Circle plot showing cell-cell interactions via APRIL signaling in the ON and YN groups.
E. Network plots showing the changes in ligand-receptor interaction events between Tregs and other immune cell types in the ON/YN comparison group. Cell-cell communication is indicated by the connected line. The thickness of the lines is positively correlated with the number of ligand-receptor interaction events.

Supplementary Fig. S7 Lymph nodes alteration during EAU, cluster of immune cells subsets, and GO analysis of the YE/YN and OE/ON comparison groups.
A. Draining lymph nodes of four groups.
B. UMAP plot showing clusters of immune cell subsets across four groups.
C. UMAP plots of canonical markers for immune cell subsets.
D. Representative GO terms and KEGG pathway enriched in downregulated DEGs of total immune cells in the YE/YN and OE/ON comparison groups.

Supplementary Fig. S8 GO analysis of immune cell subsets in the YE/YN and OE/ON comparison groups.
A-B. Representative GO terms and KEGG pathway enriched in upregulated (A) or downregulated (B) DEGs of immune cell subsets in the YE/YN and OE/ON comparison groups.

Supplementary Fig. S9 Canonical markers for T cell subsets, and GO analysis of T cell subsets in the YE/YN and OE/ON comparison groups.
A. UMAP plots of canonical markers for T cell subsets.
B. Representative GO terms and KEGG pathway enriched in downregulated DEGs of T cell subsets in the YE/YN and OE/ON comparison groups.

Supplementary Fig. S10 Re-clustering, cell ratios and GO analysis of B subsets, proportions of plasma cells and follicular T helper cells across four groups.

A. UMAP plot showing clusters of B cell subsets.

B. UMAP plots of canonical markers for B cell subsets.

C. Relative fold changes (FC) in cell ratios in B cell subsets across the four groups (YN, YE, ON, and OE). The numbers on the right indicate the Log_2FC values of the cell ratios (YE/YN, OE/ON, and OE/YE).

D-E. Representative scatter plots (D) and quantification (E) of the proportion of plasma B cells from the four groups measured by flow cytometry. Each group contains six mice. The values represent the mean ± SD from three independent experiments. Significance was determined using two-way ANOVA. ****P < 0.0001.

F-G. Representative scatter plots (F) and quantification (G) of the proportion of follicular T helper cells from the four groups were measured by flow cytometry. Each group contains six mice. The values represent the mean ± SD from three independent experiments. Significance was determined using two-way ANOVA. ****P < 0.0001.

H-I. Representative GO terms and KEGG pathway enriched in upregulated (H) or downregulated (I) DEGs of B cell subsets in the YE/YN and OE/ON comparison groups.

J. Levels of $IRBP_{1-20}$-specific antibodies in the blood serum from the four groups detected by ELISA. Each group contains six mice. The data represent the means ± SD. Significance was determined using two-way ANOVA. ****P < 0.0001. NS, not significant.

K. Concentration of total IgG in the blood serum from the four groups detected by ELISA. Each group contains six mice. The data represent the means ± SD. Significance was determined using two-way ANOVA. ****P < 0.0001.

Supplementary Fig. S11 Gating strategy of Th17 cells and schematic of the mechanism of milder EAU in aging mice.

A. Heatmap of relative expression of *Csf2* and *Il23r* from Th17 cells across the four groups.

B. Gating strategy of Th17 cells

C. Schematic illustrating the mechanism of milder EAU during aging.

Supplementary Table Legends

Supplementary Table S1. DEGs in total immune cells (A), eight major immune cell subsets (B-I), ten T cell subsets (J-S) and four B cell subsets (T-W) in the ON/YN comparison group.

Supplementary Table S2. Transcriptional factor activity of Th17 cells in the ON and YN groups.

Supplementary Table S3. DEGs in total immune cells (A), eight major immune cell subsets (B-I), ten T cell subsets (J-S) and four B cell subsets (T-W) in the YE/YN comparison group.

Supplementary Table S4. DEGs in total immune cells (A), eight major immune cell subsets (B-I), ten T cell subsets (J-S) and four B cell subsets (T-W) in the OE/ON comparison group.

Supplementary Table S5. Cell-cell interaction pairs in the YN (A), YE (B), ON (C) and OE (D) groups.

Supplementary Table S6. Clinical and pathological scoring criteria of EAU.

Figure S1

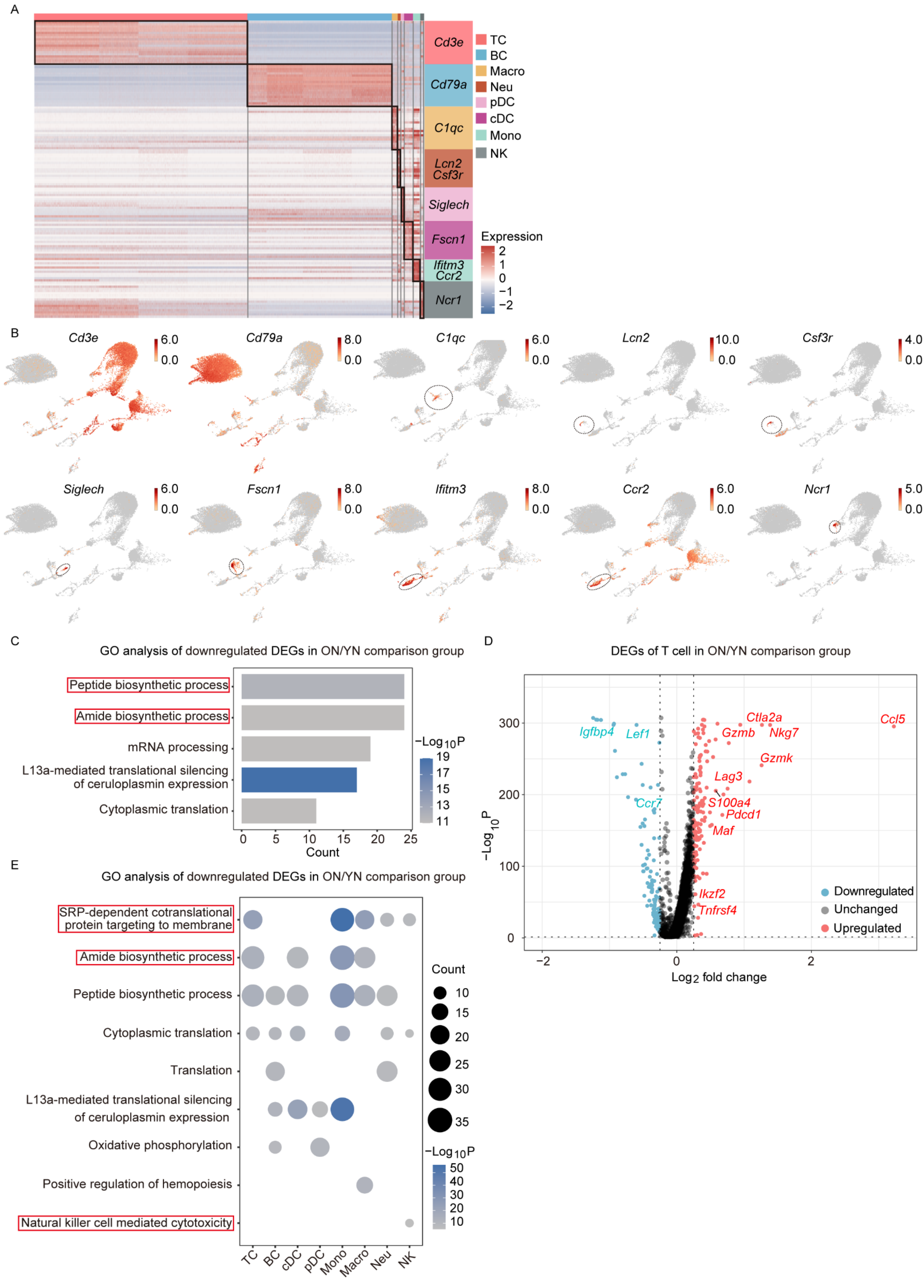
A
Cd3e
Cd79a
C1qc
Lcn2
Csf3r
Siglech
Fscn1
Ifitm3
Ccr2
Ncr1
TC
BC
Macro
Neu
pDC
cDC
Mono
NK
Expression
2
1
0
−1
−2
B
Cd3e
Cd79a
C1qc
Lcn2
Csf3r
Siglech
Fscn1
Ifitm3
Ccr2
Ncr1
C
GO analysis of downregulated DEGs in ON/YN comparison group
Peptide biosynthetic process
Amide biosynthetic process
mRNA processing
L13a-mediated translational silencing of ceruloplasmin expression
Cytoplasmic translation
Count
−Log10P
D
DEGs of T cell in ON/YN comparison group
Igfbp4
Lef1
Ccr7
Ctla2a
Gzmb
Nkg7
Ccl5
Gzmk
Lag3
S100a4
Pdcd1
Maf
Ikzf2
Tnfrsf4
Downregulated
Unchanged
Upregulated
−Log10P
Log2 fold change
E
GO analysis of downregulated DEGs in ON/YN comparison group
SRP-dependent cotranslational protein targeting to membrane
Amide biosynthetic process
Peptide biosynthetic process
Cytoplasmic translation
Translation
L13a-mediated translational silencing of ceruloplasmin expression
Oxidative phosphorylation
Positive regulation of hemopoiesis
Natural killer cell mediated cytotoxicity
Count
−Log10P
TC
BC
cDC
pDC
Mono
Macro
Neu
NK

Figure S2

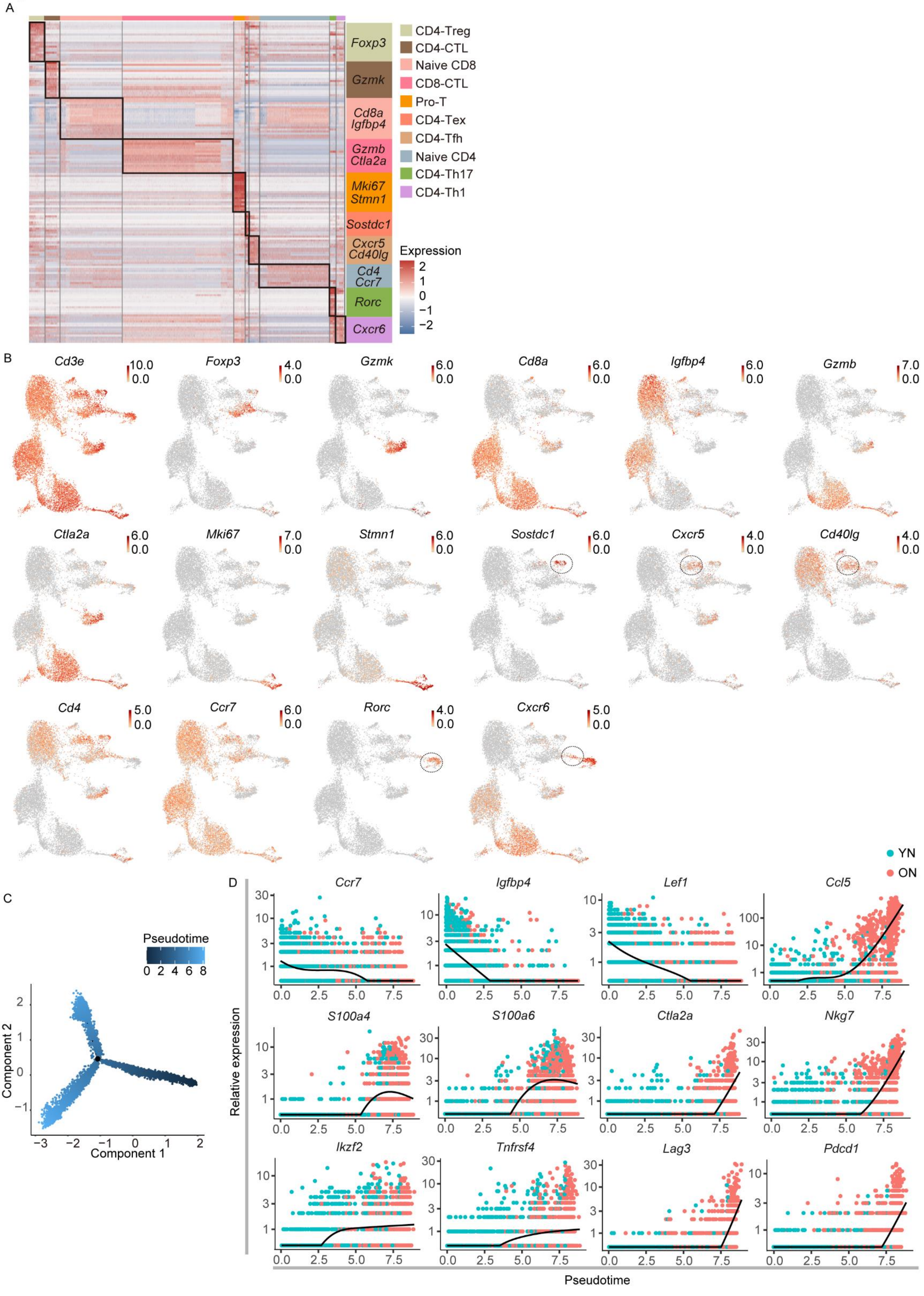
A
Foxp3
Gzmk
Cd8a Igfbp4
Gzmb Ctla2a
Mki67 Stmn1
Sostdc1
Cxcr5 Cd40lg
Cd4 Ccr7
Rorc
Cxcr6
CD4-Treg
CD4-CTL
Naive CD8
CD8-CTL
Pro-T
CD4-Tex
CD4-Tfh
Naive CD4
CD4-Th17
CD4-Th1
Expression
B
Cd3e
Foxp3
Gzmk
Cd8a
Igfbp4
Gzmb
Ctla2a
Mki67
Stmn1
Sostdc1
Cxcr5
Cd40lg
Cd4
Ccr7
Rorc
Cxcr6
C
Pseudotime
Component 1
Component 2
D
YN
ON
Ccr7
Igfbp4
Lef1
Ccl5
S100a4
S100a6
Ctla2a
Nkg7
Ikzf2
Tnfrsf4
Lag3
Pdcd1
Relative expression
Pseudotime

Figure S3

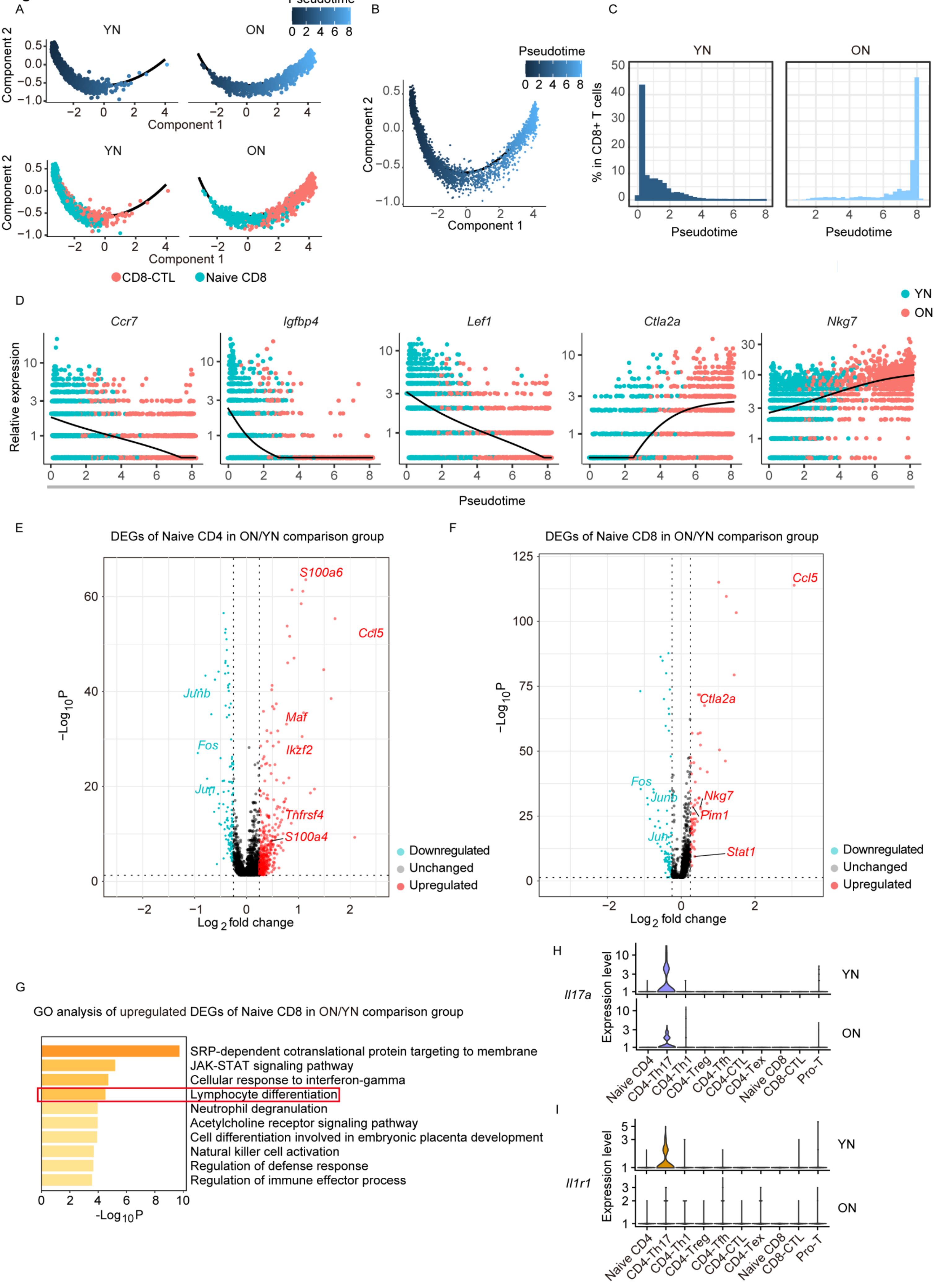
A
Pseudotime
YN
ON
Component 2
Component 1
CD8-CTL
Naive CD8
B
C
% in CD8+ T cells
Pseudotime
D
YN
ON
Ccr7
Igfbp4
Lef1
Ctla2a
Nkg7
Relative expression
Pseudotime
E
DEGs of Naive CD4 in ON/YN comparison group
S100a6
Ccl5
Junb
Maf
Ikzf2
Fos
Jun
Tnfrsf4
S100a4
-Log10P
Log 2 fold change
Downregulated
Unchanged
Upregulated
F
DEGs of Naive CD8 in ON/YN comparison group
Ccl5
Ctla2a
Fos
Junb
Nkg7
Pim1
Jun
Stat1
G
GO analysis of upregulated DEGs of Naive CD8 in ON/YN comparison group
SRP-dependent cotranslational protein targeting to membrane
JAK-STAT signaling pathway
Cellular response to interferon-gamma
Lymphocyte differentiation
Neutrophil degranulation
Acetylcholine receptor signaling pathway
Cell differentiation involved in embryonic placenta development
Natural killer cell activation
Regulation of defense response
Regulation of immune effector process
-Log10P
H
Il17a
Expression level
Naive CD4
CD4-Th17
CD4-Th1
CD4-Treg
CD4-Tfh
CD4-CTL
CD4-Tex
Naive CD8
CD8-CTL
Pro-T
I
Il1r1

Figure S4

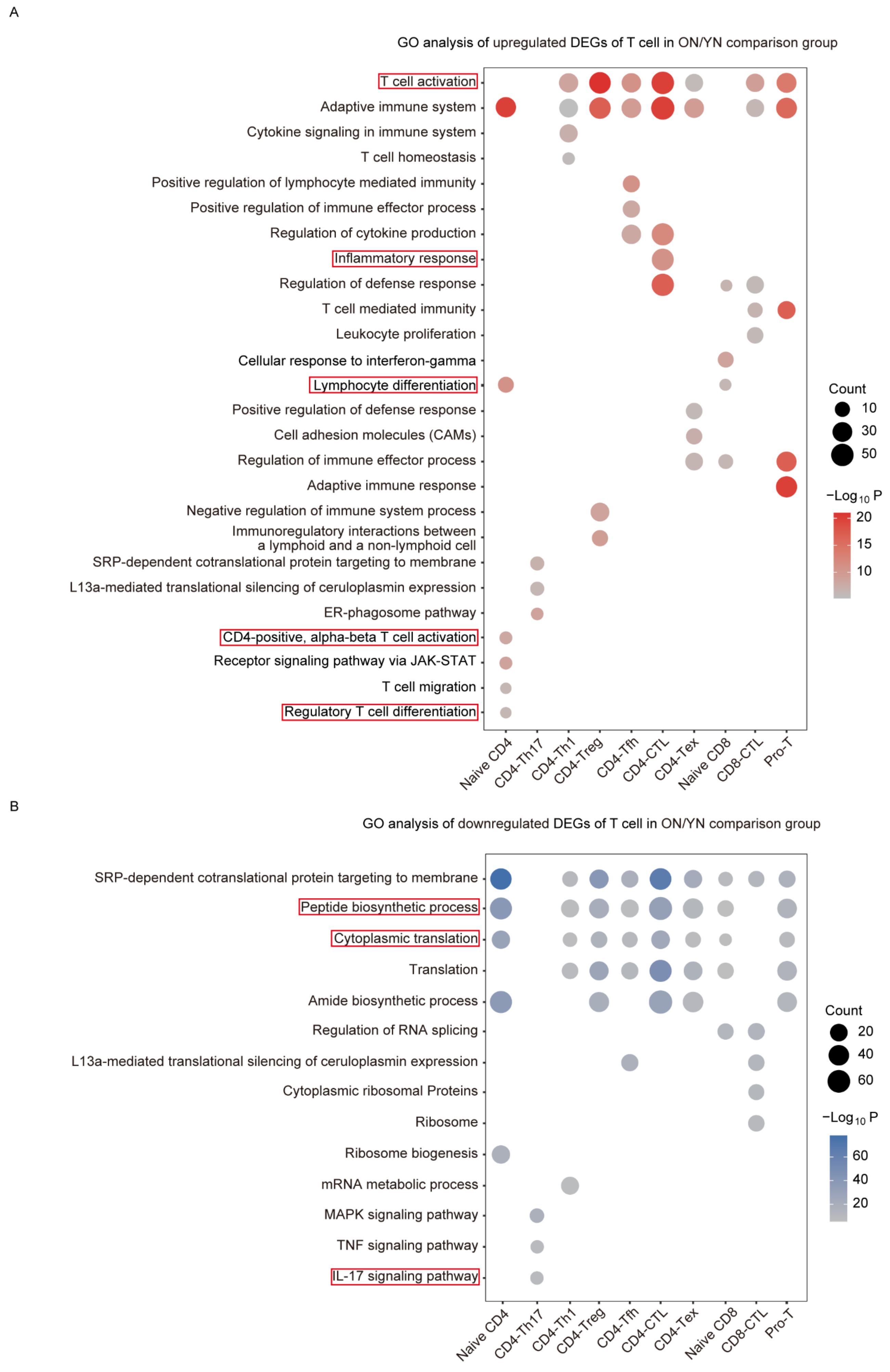
A
GO analysis of upregulated DEGs of T cell in ON/YN comparison group
T cell activation
Adaptive immune system
Cytokine signaling in immune system
T cell homeostasis
Positive regulation of lymphocyte mediated immunity
Positive regulation of immune effector process
Regulation of cytokine production
Inflammatory response
Regulation of defense response
T cell mediated immunity
Leukocyte proliferation
Cellular response to interferon-gamma
Lymphocyte differentiation
Positive regulation of defense response
Cell adhesion molecules (CAMs)
Regulation of immune effector process
Adaptive immune response
Negative regulation of immune system process
Immunoregulatory interactions between a lymphoid and a non-lymphoid cell
SRP-dependent cotranslational protein targeting to membrane
L13a-mediated translational silencing of ceruloplasmin expression
ER-phagosome pathway
CD4-positive, alpha-beta T cell activation
Receptor signaling pathway via JAK-STAT
T cell migration
Regulatory T cell differentiation
Naive CD4
CD4-Th17
CD4-Th1
CD4-Treg
CD4-Tfh
CD4-CTL
CD4-Tex
Naive CD8
CD8-CTL
Pro-T
Count
10
30
50
−Log10 P
20
15
10
B
GO analysis of downregulated DEGs of T cell in ON/YN comparison group
SRP-dependent cotranslational protein targeting to membrane
Peptide biosynthetic process
Cytoplasmic translation
Translation
Amide biosynthetic process
Regulation of RNA splicing
L13a-mediated translational silencing of ceruloplasmin expression
Cytoplasmic ribosomal Proteins
Ribosome
Ribosome biogenesis
mRNA metabolic process
MAPK signaling pathway
TNF signaling pathway
IL-17 signaling pathway
Naive CD4
CD4-Th17
CD4-Th1
CD4-Treg
CD4-Tfh
CD4-CTL
CD4-Tex
Naive CD8
CD8-CTL
Pro-T
Count
20
40
60
−Log10 P
60
40
20

Figure S5

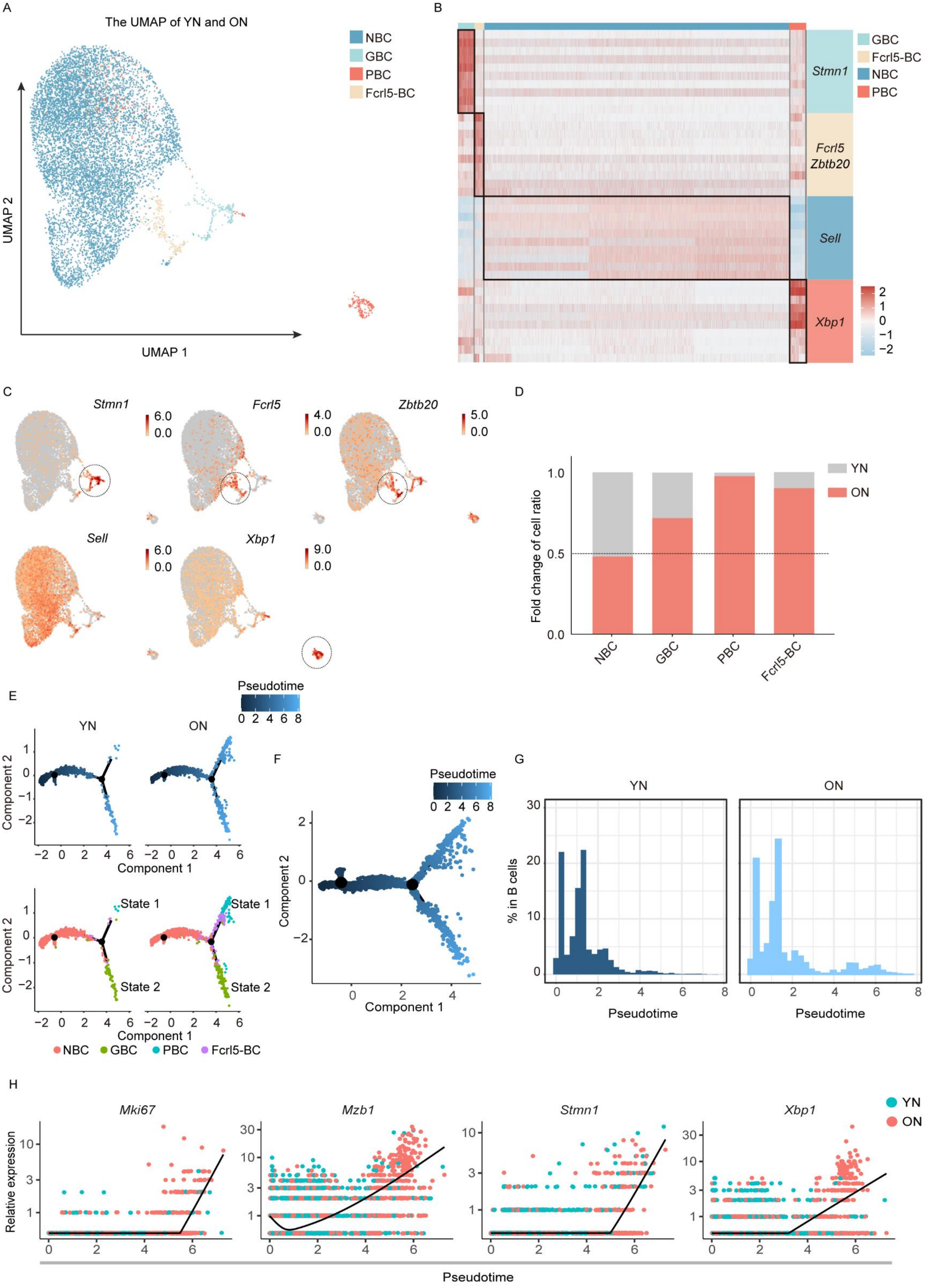
A
The UMAP of YN and ON
UMAP 1
UMAP 2
NBC
GBC
PBC
Fcrl5-BC
B
GBC
Fcrl5-BC
NBC
PBC
Stmn1
Fcrl5
Zbtb20
Sell
Xbp1
C
Stmn1
Fcrl5
Zbtb20
Sell
Xbp1
D
Fold change of cell ratio
YN
ON
NBC
GBC
PBC
Fcrl5-BC
E
Pseudotime
YN
ON
Component 1
Component 2
State 1
State 2
NBC
GBC
PBC
Fcrl5-BC
F
Pseudotime
Component 1
Component 2
G
YN
ON
% in B cells
Pseudotime
H
Mki67
Mzb1
Stmn1
Xbp1
YN
ON
Relative expression
Pseudotime

Figure S6

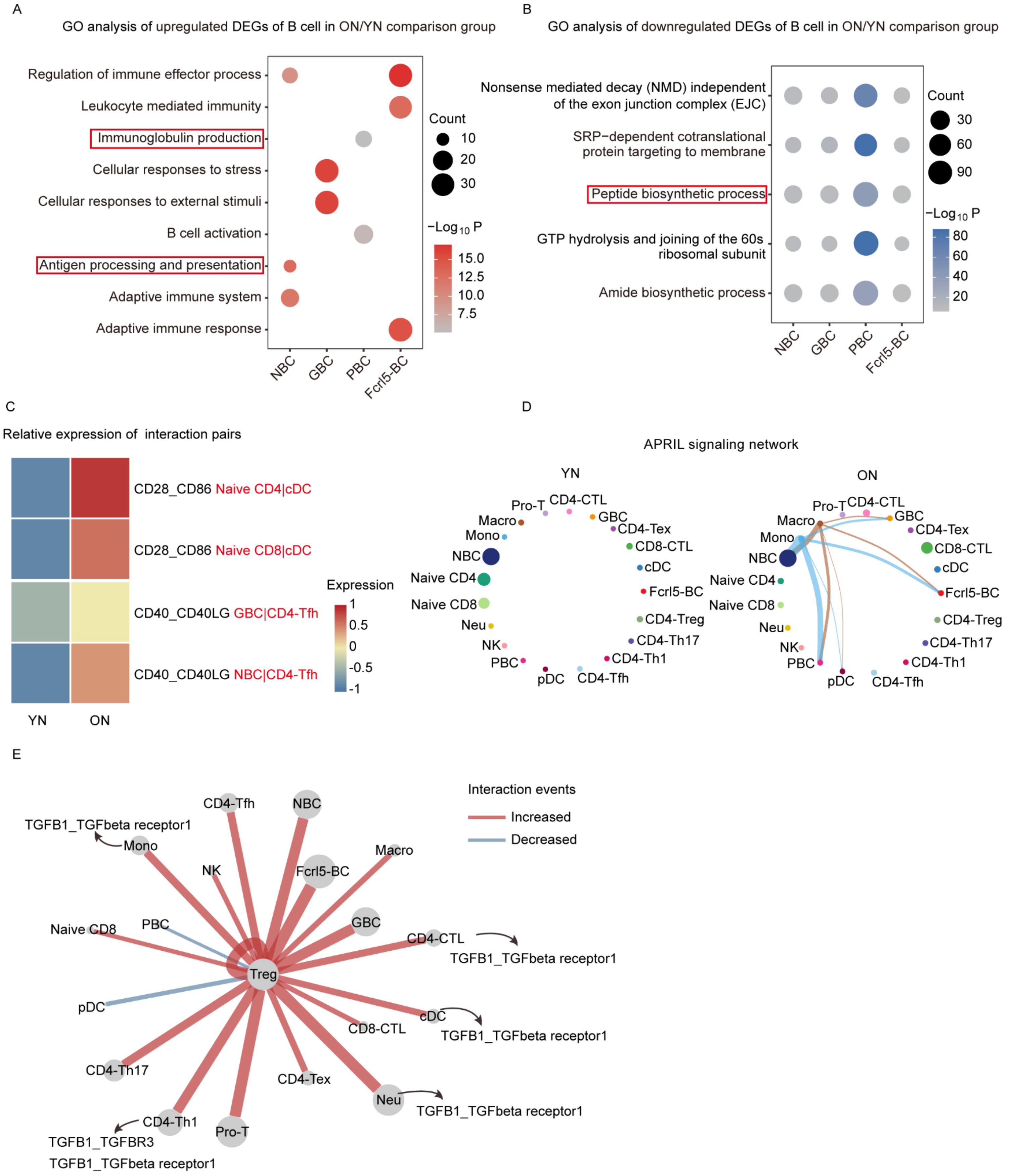
A
GO analysis of upregulated DEGs of B cell in ON/YN comparison group
Regulation of immune effector process
Leukocyte mediated immunity
Immunoglobulin production
Cellular responses to stress
Cellular responses to external stimuli
B cell activation
Antigen processing and presentation
Adaptive immune system
Adaptive immune response
NBC
GBC
PBC
Fcrl5-BC
Count
10
20
30
−Log10 P
15.0
12.5
10.0
7.5
B
GO analysis of downregulated DEGs of B cell in ON/YN comparison group
Nonsense mediated decay (NMD) independent of the exon junction complex (EJC)
SRP−dependent cotranslational protein targeting to membrane
Peptide biosynthetic process
GTP hydrolysis and joining of the 60s ribosomal subunit
Amide biosynthetic process
NBC
GBC
PBC
Fcrl5-BC
Count
30
60
90
−Log10 P
80
60
40
20
C
Relative expression of interaction pairs
CD28_CD86 Naive CD4|cDC
CD28_CD86 Naive CD8|cDC
CD40_CD40LG GBC|CD4-Tfh
CD40_CD40LG NBC|CD4-Tfh
Expression
1
0.5
0
-0.5
-1
YN
ON
D
APRIL signaling network
YN
ON
Pro-T
CD4-CTL
GBC
CD4-Tex
CD8-CTL
cDC
Fcrl5-BC
CD4-Treg
CD4-Th17
CD4-Th1
CD4-Tfh
pDC
PBC
NK
Neu
Naive CD8
Naive CD4
NBC
Mono
Macro
E
Interaction events
Increased
Decreased
TGFB1_TGFbeta receptor1
Mono
CD4-Tfh
NBC
NK
Fcrl5-BC
Macro
Naive CD8
PBC
GBC
CD4-CTL
Treg
pDC
cDC
CD8-CTL
CD4-Th17
CD4-Tex
Neu
CD4-Th1
Pro-T
TGFB1_TGFBR3
TGFB1_TGFbeta receptor1

Figure S7

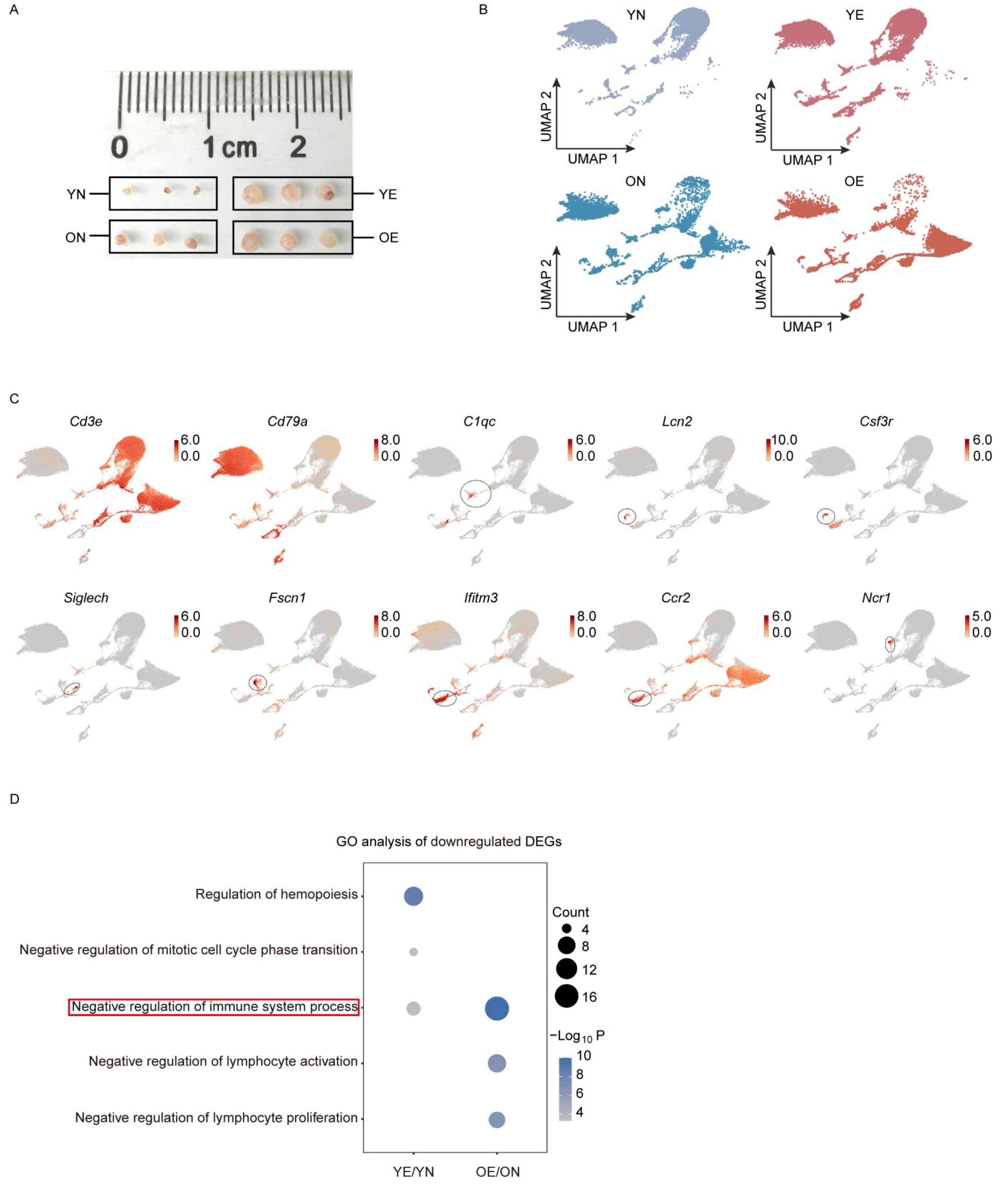
A
0
1 cm
2
YN
YE
ON
OE
B
YN
YE
ON
OE
UMAP 2
UMAP 1
C
Cd3e
Cd79a
C1qc
Lcn2
Csf3r
Siglech
Fscn1
Ifitm3
Ccr2
Ncr1
6.0
8.0
10.0
5.0
0.0
D
GO analysis of downregulated DEGs
Regulation of hemopoiesis
Negative regulation of mitotic cell cycle phase transition
Negative regulation of immune system process
Negative regulation of lymphocyte activation
Negative regulation of lymphocyte proliferation
Count
4
8
12
16
−Log10 P
10
8
6
4
YE/YN
OE/ON

Figure S8

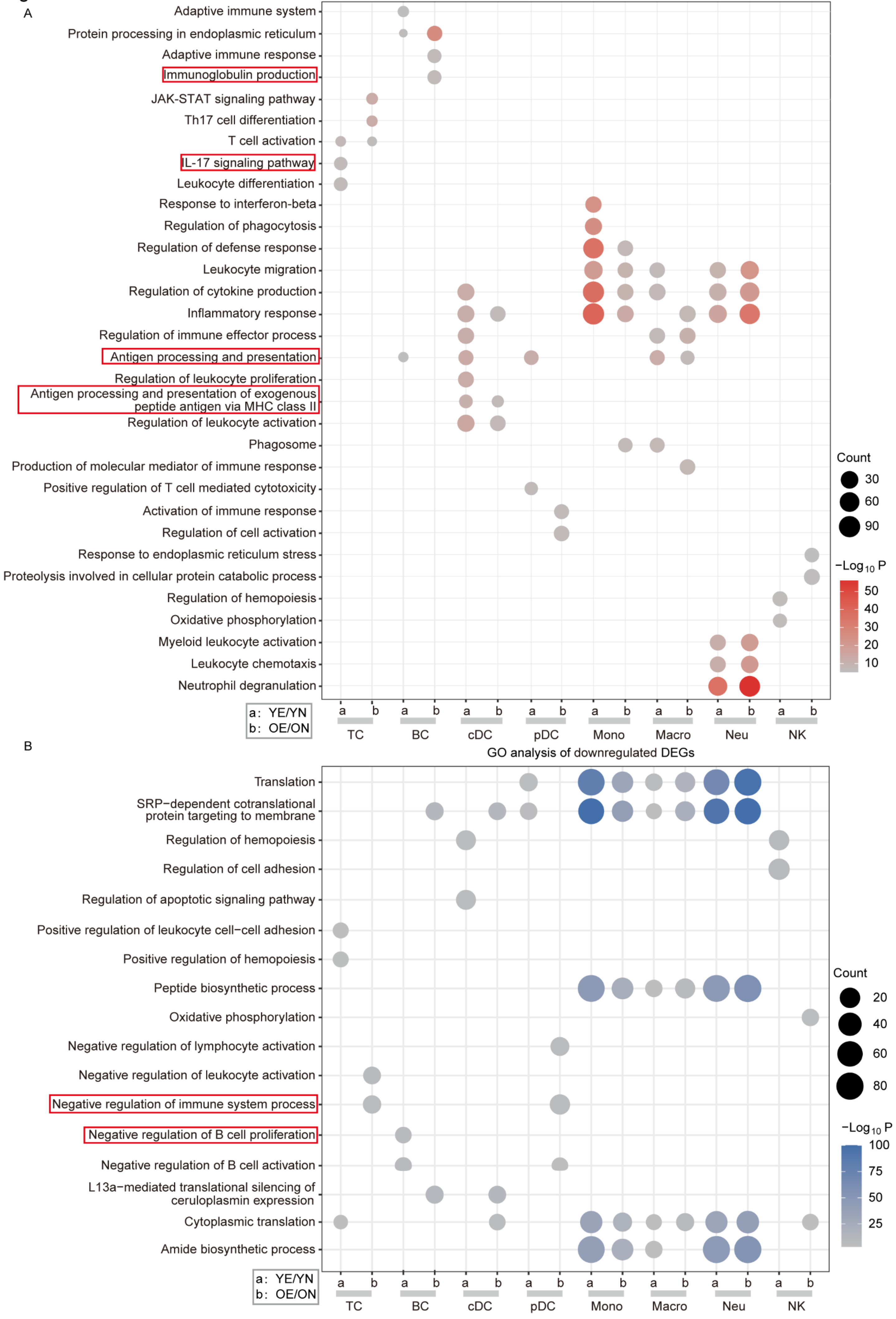
A
GO analysis of upregulated DEGs
Adaptive immune system
Protein processing in endoplasmic reticulum
Adaptive immune response
Immunoglobulin production
JAK-STAT signaling pathway
Th17 cell differentiation
T cell activation
IL-17 signaling pathway
Leukocyte differentiation
Response to interferon-beta
Regulation of phagocytosis
Regulation of defense response
Leukocyte migration
Regulation of cytokine production
Inflammatory response
Regulation of immune effector process
Antigen processing and presentation
Regulation of leukocyte proliferation
Antigen processing and presentation of exogenous peptide antigen via MHC class II
Regulation of leukocyte activation
Phagosome
Production of molecular mediator of immune response
Positive regulation of T cell mediated cytotoxicity
Activation of immune response
Regulation of cell activation
Response to endoplasmic reticulum stress
Proteolysis involved in cellular protein catabolic process
Regulation of hemopoiesis
Oxidative phosphorylation
Myeloid leukocyte activation
Leukocyte chemotaxis
Neutrophil degranulation
a: YE/YN
b: OE/ON
TC
BC
cDC
pDC
Mono
Macro
Neu
NK
Count
30
60
90
−Log10 P
50
40
30
20
10
B
GO analysis of downregulated DEGs
Translation
SRP−dependent cotranslational protein targeting to membrane
Regulation of hemopoiesis
Regulation of cell adhesion
Regulation of apoptotic signaling pathway
Positive regulation of leukocyte cell−cell adhesion
Positive regulation of hemopoiesis
Peptide biosynthetic process
Oxidative phosphorylation
Negative regulation of lymphocyte activation
Negative regulation of leukocyte activation
Negative regulation of immune system process
Negative regulation of B cell proliferation
Negative regulation of B cell activation
L13a−mediated translational silencing of ceruloplasmin expression
Cytoplasmic translation
Amide biosynthetic process
a: YE/YN
b: OE/ON
TC
BC
cDC
pDC
Mono
Macro
Neu
NK
Count
20
40
60
80
−Log10 P
100
75
50
25

Figure S9

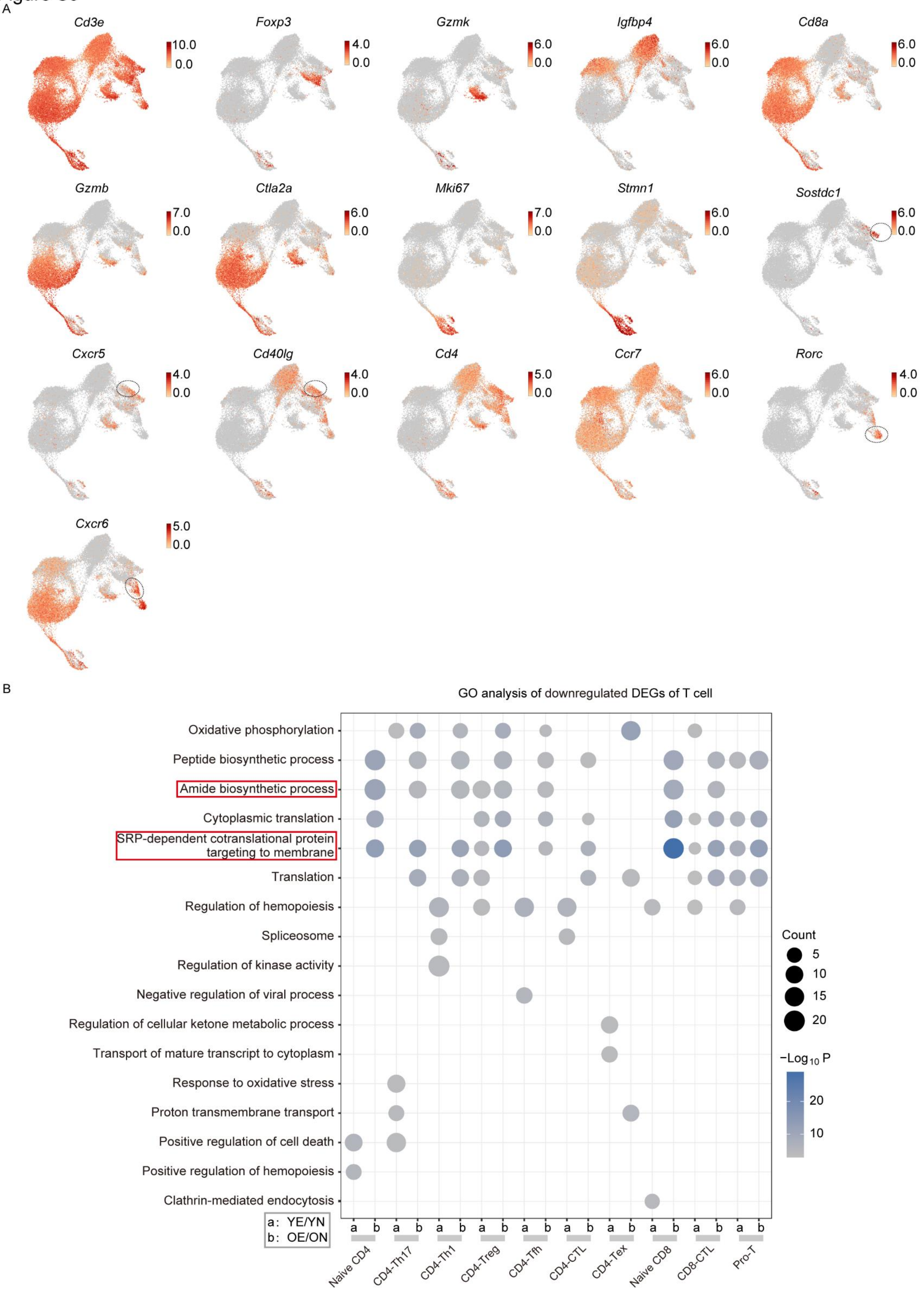
A
Cd3e
Foxp3
Gzmk
Igfbp4
Cd8a
Gzmb
Ctla2a
Mki67
Stmn1
Sostdc1
Cxcr5
Cd40lg
Cd4
Ccr7
Rorc
Cxcr6
B
GO analysis of downregulated DEGs of T cell
Oxidative phosphorylation
Peptide biosynthetic process
Amide biosynthetic process
Cytoplasmic translation
SRP-dependent cotranslational protein targeting to membrane
Translation
Regulation of hemopoiesis
Spliceosome
Regulation of kinase activity
Negative regulation of viral process
Regulation of cellular ketone metabolic process
Transport of mature transcript to cytoplasm
Response to oxidative stress
Proton transmembrane transport
Positive regulation of cell death
Positive regulation of hemopoiesis
Clathrin-mediated endocytosis
a: YE/YN
b: OE/ON
Naive CD4
CD4-Th17
CD4-Th1
CD4-Treg
CD4-Tfh
CD4-CTL
CD4-Tex
Naive CD8
CD8-CTL
Pro-T
Count
5
10
15
20
−Log10 P
20
10

Figure S10

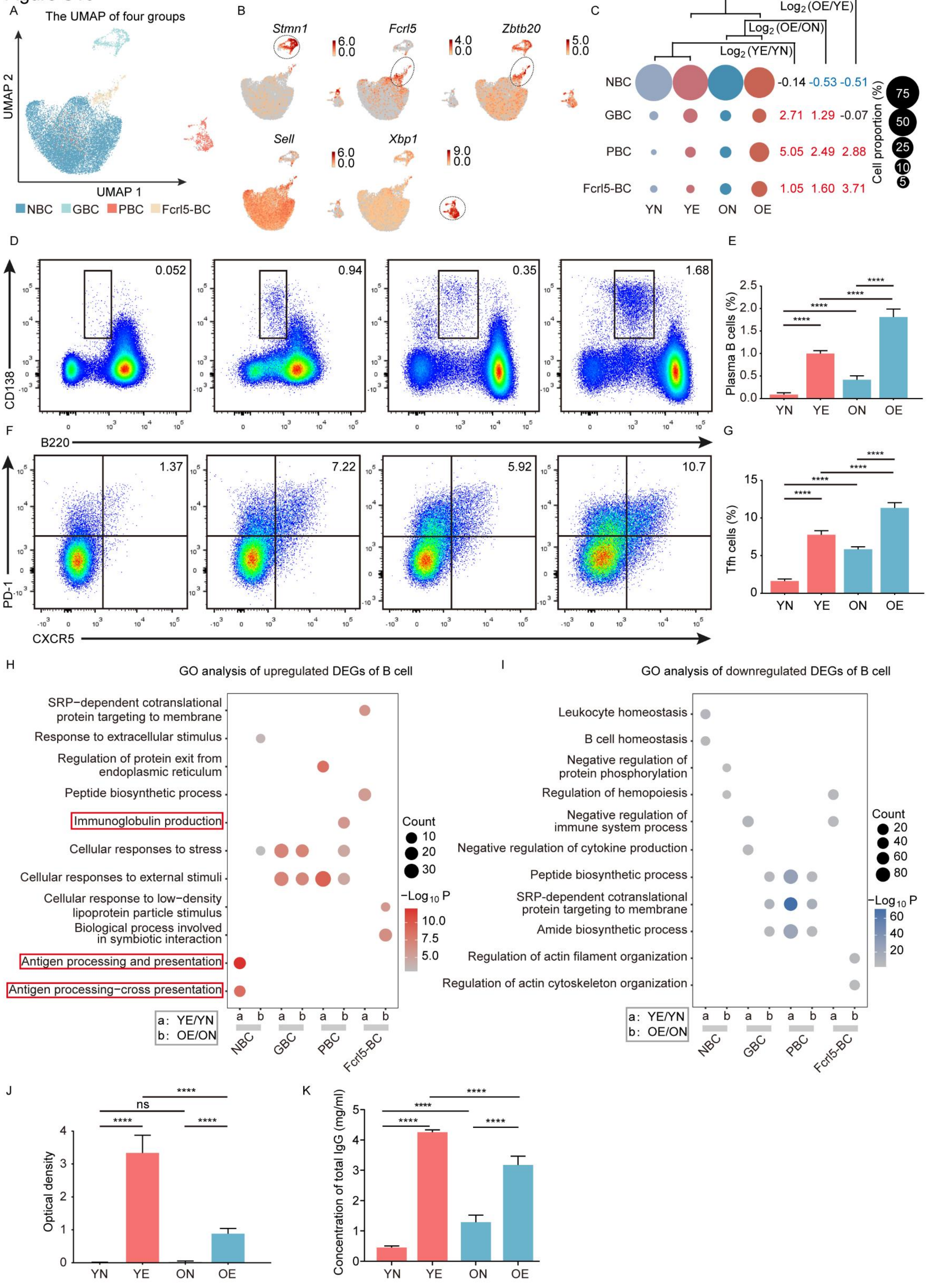

A
The UMAP of four groups
UMAP 2
UMAP 1
NBC GBC PBC Fcrl5-BC
B
Stmn1
Fcrl5
Zbtb20
Sell
Xbp1
C
Log2 (OE/YE)
Log2 (OE/ON)
Log2 (YE/YN)
NBC -0.14 -0.53 -0.51
GBC 2.71 1.29 -0.07
PBC 5.05 2.49 2.88
Fcrl5-BC 1.05 1.60 3.71
YN YE ON OE
Cell proportion (%)
D
0.052
0.94
0.35
1.68
CD138
B220
E
Plasma B cells (%)
F
1.37
7.22
5.92
10.7
PD-1
CXCR5
G
Tfh cells (%)
H
GO analysis of upregulated DEGs of B cell
SRP−dependent cotranslational protein targeting to membrane
Response to extracellular stimulus
Regulation of protein exit from endoplasmic reticulum
Peptide biosynthetic process
Immunoglobulin production
Cellular responses to stress
Cellular responses to external stimuli
Cellular response to low−density lipoprotein particle stimulus
Biological process involved in symbiotic interaction
Antigen processing and presentation
Antigen processing−cross presentation
Count
−Log10 P
a: YE/YN
b: OE/ON
NBC GBC PBC Fcrl5-BC
I
GO analysis of downregulated DEGs of B cell
Leukocyte homeostasis
B cell homeostasis
Negative regulation of protein phosphorylation
Regulation of hemopoiesis
Negative regulation of immune system process
Negative regulation of cytokine production
Peptide biosynthetic process
SRP-dependent cotranslational protein targeting to membrane
Amide biosynthetic process
Regulation of actin filament organization
Regulation of actin cytoskeleton organization
Count
−Log10 P
a: YE/YN
b: OE/ON
NBC GBC PBC Fcrl5-BC
J
Optical density
YN YE ON OE
K
Concentration of total IgG (mg/ml)
YN YE ON OE

Figure S11

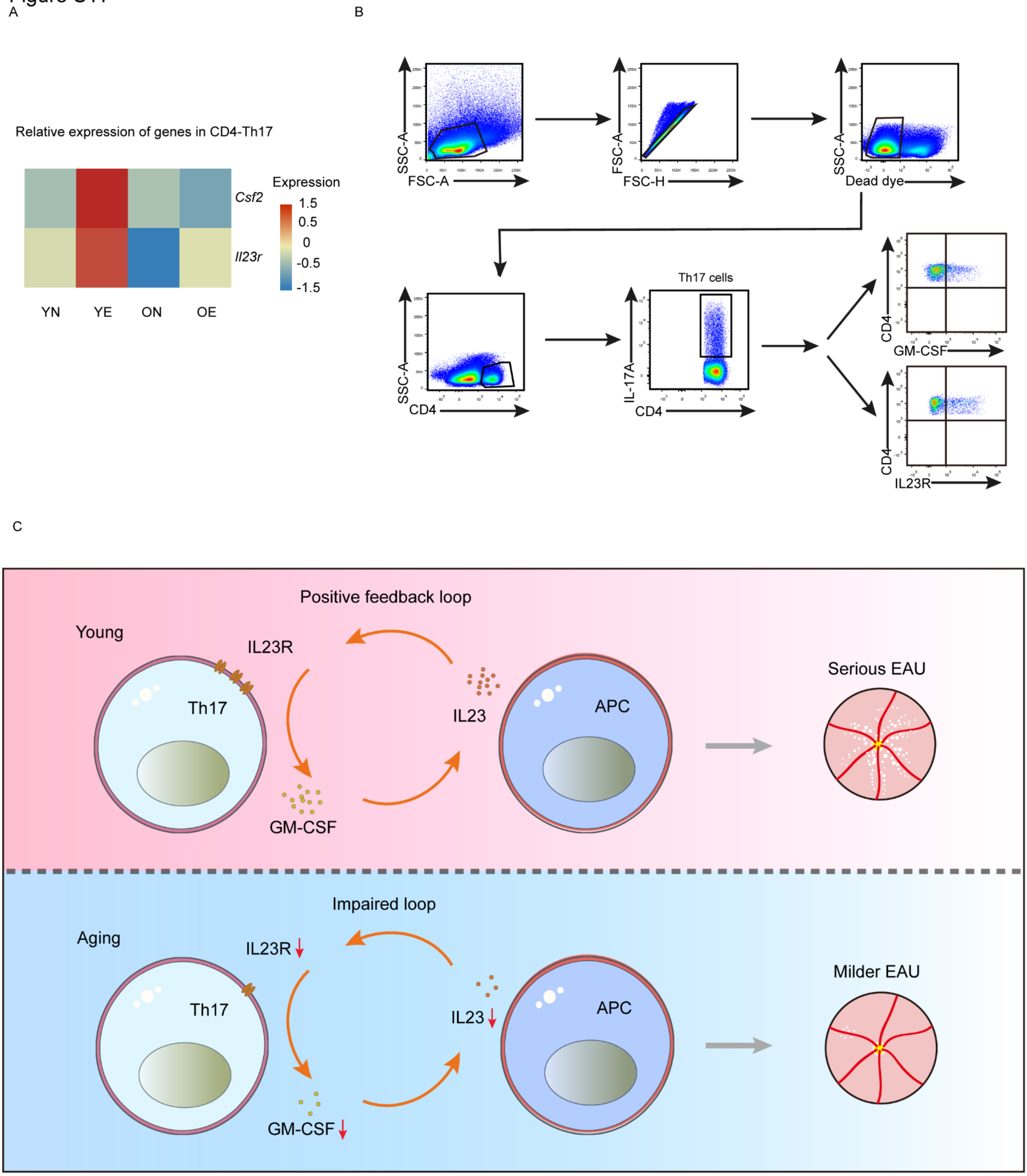
A
B
Relative expression of genes in CD4-Th17
Expression
1.5
0.5
0
-0.5
-1.5
Csf2
Il23r
YN
YE
ON
OE
SSC-A
FSC-A
FSC-H
Dead dye
Th17 cells
IL-17A
CD4
GM-CSF
IL23R
C
Positive feedback loop
Young
IL23R
Th17
IL23
APC
GM-CSF
Serious EAU
Impaired loop
Aging
IL23R
Th17
IL23
APC
GM-CSF
Milder EAU
